# Global Gene Expression of T Cells Is Differentially Regulated by Peritoneal Dendritic Cell Subsets in an IL-2 Dependent Manner

**DOI:** 10.3389/fimmu.2021.648348

**Published:** 2021-05-17

**Authors:** Moah Sohn, Hye Young Na, Hyun Soo Shin, Seul Hye Ryu, Sejung Park, Hyunju In, Wanho Choi, Ji Soo Park, Soomin Hwang, Min Kyung Chu, Chae Gyu Park

**Affiliations:** ^1^ Laboratory of Immunology, Severance Biomedical Science Institute, Yonsei University College of Medicine, Seoul, South Korea; ^2^ Brain Korea 21 FOUR Project for Medical Science, Yonsei University College of Medicine, Seoul, South Korea; ^3^ Department of Neurology, Severance Hospital, Yonsei University College of Medicine, Seoul, South Korea; ^4^ Therapeutic Antibody Research Center, GENUV Inc., Seoul, South Korea; ^5^ Institute for Immunology and Immunological Diseases, Yonsei University College of Medicine, Seoul, South Korea

**Keywords:** antigen presentation, dendritic cell, interleukin-2, peritoneal cavity, T cell – DC interactions

## Abstract

Dendritic cells (DCs) in peripheral tissues may have a unique role to regulate innate and adaptive immune responses to antigens that enter the tissues. Peritoneal cavity is the body compartment surrounding various tissues and organs and housing diverse immune cells. Here, we investigated the specialized features of classical DC (cDC) subsets following the intraperitoneal injection of a model antigen ovalbumin (OVA). Peritoneal cDC1s were superior to cDC2s in activating OVA-specific CD8 T cells, while both cDCs were similar in stimulating OVA-specific CD4 T cells. Each peritoneal cDC subset differentially regulated the homing properties of CD8 T cells. CD8 T cells stimulated by cDC1s displayed a higher level of lung-homing receptor CCR4, whereas those stimulated by cDC2s prominently expressed various homing receptors including gut-homing molecules CCR9 and α4β7. Also, we found that cDC1s played a dominating role over cDC2s in controlling the overall gene expression of CD8 T cells. Soluble factor(s) emanating from CD8 T cells stimulated by peritoneal cDC1s were responsible for mediating this dominance of cDC1s, and we identified IL-2 as a soluble factor regulating the global gene expression of T cells. Collectively, our study indicates that different peritoneal cDC subsets effectively diversify T cell responses by altering the level of cytokines, such as IL-2, in the milieu.

## Introduction

Dendritic cells (DCs) are professional antigen-presenting cells (APCs) that orchestrate innate and adaptive immune responses (1, 2). DCs are a heterogeneous group of cells with various subsets that are differentiated by their ontogeny, localization, and immunological functions (3). DCs can be divided into 3 major subsets, i.e., classical DCs (cDCs) processing and presenting antigens (Ags) in the steady state, monocyte-derived DCs developing and functioning under inflammatory conditions, and plasmacytoid DCs producing type I interferon to facilitate anti-viral immune responses. Again, cDCs are further divided into at least 2 main subsets, cDC1s and cDC2s. cDC1s are better at cross-presenting Ags to induce CD8 T cell responses, and cDC2s are efficient at promoting CD4 T cells to proliferate and differentiate (3). Distinct DC subsets have been further identified in various tissues under different conditions, and are shown to carry out specialized functions (4–10). Meanwhile, recent studies have compared the transcriptomes of DC subsets in various tissues and illustrated the commonalities and differences in their expression of genes across tissues (11, 12), which further suggests that DC subsets residing in particular tissues may possess unique abilities and functions in addition to the general features of DCs.

DCs regulate and determine the type of T cell responses with multiple signals. DCs process Ags and present them to T cells in the form of MHC-bound peptides. The responses of cognate T cells are further modulated by the interaction of co-stimulatory molecules on the surface of T cells and DCs, and additionally by the cytokine milieu (13). IL-2 is one of the important cytokines that regulate the key aspects of T cell biology (14). IL-2 enhances immunostimulatory responses by promoting CD4 T helper type 1 and type 2 cells as well as CD8 cytotoxic T (Tc) cells, yet it also contributes to immunosuppressive responses by boosting the development and function of regulatory T cells (14–17). Besides, IL-2 also affects the durability of immune responses. Weak or limited IL-2 signaling is critical in the formation of memory T cells (18, 19), whereas strong IL-2 signaling, in combination with inflammatory cytokines, induces the expression of transcription factors T-bet and Blimp-1, which in turn promotes the differentiation of short-lived CD8 Tc cells (20). Therefore, the regulation of IL-2 in the milieu is important in dictating the fate of T cells. Although it is believed that DCs have an important role in modulating various cytokines in the milieu and thus regulating the outcome of T cell responses (21–23), it has remained poorly understood how and which DC subsets are involved in controlling IL-2 in the milieu.

The peritoneal cavity is a specialized compartment enclosed by the peritoneum that houses multiple tissues and immune cells. Macrophages and B1 cells constitute the majority of peritoneal immune cells, and others such as T cells, eosinophils, neutrophils, NK cells, and DCs are found in smaller numbers (24). Since the peritoneum is supplied with abundant blood vessels, the peritoneal cavity is being commonly used as a systemic injection route for small laboratory animals. Following the intraperitoneal (i.p.) injection, peritoneal DCs must be responsible for the capture, process, and presentation of Ags to T cells and thus initiating immune responses; however, studies regarding those aspects of peritoneal DCs have been limited. Although recent studies have characterized the diversity of peritoneal DC subsets, the distinct roles between peritoneal cDC subsets are yet to be investigated in depth (25–27). In the previous study (27), we demonstrated that new subsets of peritoneal DCs and macrophages were identified by differences in their ability to present antigens to and thus stimulate T cells. Our present study examines and compares the capabilities of peritoneal cDC subsets regarding their ability to control T cell responses, and we have discovered that peritoneal cDC1s have a dominating role in modulating CD8 T cell responses by upregulating the expression of IL-2.

## Materials and Methods

### Mice

C57BL/6J mice were purchased from Orient Bio (Seongnam, Korea) and female mice between 6 and 12 weeks of age were used for the experiments. C57BL/6-Tg(TcraTcrb)1100Mjb/J (OT-1), B6.Cg-Tg(TcraTcrb)425Cbn/J (OT-2), and B6.SJL-Ptprc^a^Pepc^b^/BoyJ (CD45.1) mice were obtained from the Jackson Laboratory (Bar Harbor, ME, USA). CD45.1^+^ OT-1 mice were bred in house. All mice were bred and maintained in specific pathogen-free facilities in accordance with the guidelines approved by the Institutional Animal Care and Use Committees of the Yonsei University College of Medicine.

### Tissue Harvest and Cell Preparation

Peritoneal exudate cells were isolated following the protocols previously described (27). In brief, 5 ml of ice-cold isolation buffer, DPBS containing 3% fetal bovine serum, was injected into the peritoneal cavity. The peritoneal cavity was then gently massaged to dislodge the attached immune cells and the suspended peritoneal cells were harvested. The collected cell suspension was passed through a 100 µm strainer, and the cells were washed twice and used for experiments.

For spleen cell preparation, the spleen was mechanically disrupted with a cell strainer and syringe plunger. Disrupted cells were then washed twice with DPBS to filter fibrous materials through the strainer. Red blood cells (RBCs) were lysed with ACKlysis solution (BioLegend, San Diego, CA, USA) and washed twice with DPBS before use (28).

For lymph node single-cell preparation, lymph nodes were isolated and mechanically disrupted on a 100 µm cell strainer with a syringe plunger. Fibrous materials were filtered again with a strainer before use (29).

The isolation method of intestinal cells was modified from protocols previously described (30). In brief, small and large intestines were removed and placed in cold DMEM containing 5% FBS (Avantor Seradigm, Randor, USA). Mesentery lymph nodes and Peyer’s patches were carefully removed. Intestines were opened longitudinally and cut into 5 cm pieces and fecal content was washed. Washed intestinal tissues were incubated with 1 mM EDTA in DMEM at 37°C for 15 minutes stirred at 250 rpm. The supernatant containing intestinal epithelial cells (IEC) was collected and used. To isolate the lamina propria lymphocytes (LPL), the remaining intestinal tissue was washed in DMEM containing 5% FBS, minced, resuspended in 20 ml of DMEM containing 1 mg/ml of collagenase D (Roche, Basel, Switzerland), 500 μg/ml of dispase, 50 μg/ml of DNase (Roche), and 1% FBS (Avantor Seradigm), and was stirred at 250 rpm for 35 minutes at 37°C. The tissue suspension was collected and passed through a 100 µm cell strainer, and remaining tissue fragments were further disrupted mechanically by rubbing with a syringe plunger. Collected lamina propria cells were washed twice before use.

For lung cell preparation, the pulmonary circulation was perfused with 10 ml of HBSS to remove the intravascular pool of cells. Lungs were separated, minced thoroughly, and incubated for 35 minutes in a digestion media containing 5 mg of collagenase D (Roche) at 37°C. In the last 5 minutes, 10 mM EDTA was added to quench the digesting action. Tissue fragments were further disrupted mechanically on 100 µm cell strainers by rubbing with a syringe plunger. The tissue suspension passed through strainers was washed with HBSS then RBCs were lysed with ACKlysis solution. Cells were washed twice with HBSS before use (29).

### Antibodies and Reagents

The following reagents were purchased from BioLegend: anti-Ly6G (1A8), anti-CD3 (17A2), anti-CD19 (6D5), anti-CD115 (AFS98), anti-XCR1 (ZET), anti-CD11c (N418), anti-I-A/I-E (anti-MHC II, M5/114.15.2), anti-CD45.1 (A20), anti-CD45.2 (104), anti-CD4 (GK1.5), anti-CD8a (53-5.8), anti-Vα2 (B20.1), anti-TCRβ chain (H57-597), anti-CD11b (M1/70), anti-F4/80 (BM8), anti-B220 (RA3-6B2), anti- α4β7 (DATK32), anti-CCR9 (L053E8), anti-CD69 (H1.2F3), anti-CD25 (anti-IL-2Rα, PC61, unless indicated otherwise), anti-CCR4 (2G12), anti-CD44 (IM7), anti-IL-2Rβ (TM-β1), anti-CD49b (pan-NK cells, DX5), recombinant mouse IL-6, recombinant mouse IL-2, anti-IL-2 (JES6-1A12), anti-SIINFEKL peptide bound to mouse H-2K^b^ (25-D1.16), anti-CD14 (Sa14-2), anti-CD40 (3/23), anti-CD80 (16-10A1), anti-CD83 (Michel-19), anti-CD86 (GL-1), anti-PD-L1 (10F.9G2), anti-PD-L2 (TY25), anti-OX40L (RM134L), anti-H-2 (M1/42), and brefeldin A. Affinity purified polyclonal anti-IL-2 was purchased from R&D systems (Minneapolis, MN, USA). LPS and all trans-retinoic acid were purchased from Sigma-Aldrich and human TGF-β1 from Peprotech (Rocky Hill, NJ, USA). Cell Trace™ CFSE cell proliferation kit (Thermo Fisher Scientific, Waltham, MA, USA), Cell Trace™ Violet cell proliferation kit (Thermo Fisher Scientific), LIVE/DEAD^®^ fixable dead cell stain kit (Thermo Fisher Scientific) were purchased and used according to the manufacturer’s instructions. Grade V OVA was purchased from Sigma-Aldrich (St. Louis, MO, USA) and Endofit OVA was obtained from InvivoGen (San Diego, CD, USA).

### Flow Cytometric Analysis and Intracellular Cytokine Staining

Cells were isolated as described in the previous section. To block nonspecific staining, isolated single cell suspensions were incubated with the culture supernatant of 2.4G2 (anti-CD16/32) monoclonal antibody hybridoma for 20 minutes at 4°C. Cells were washed with FACS buffer (DPBS containing 2% of FBS and 2 mM EDTA), then antibodies for cell surface markers and dead cell staining dyes were added and incubated for 20 minutes at 4°C. After surface staining, cells were washed twice and analyzed or sorted with flow cytometry. For intracellular staining, cells were incubated with brefeldin A for the last 4 hours before being harvested. Cells were processed for surface staining. Then, surface-stained cells were resuspended in Fixation/Permeabilization solution (BioLegend) and intracellular cytokine staining was performed according to the manufacturer’s protocol. FACSVerse™ and LSRFortessa™ flow cytometers (BD Biosciences, San Jose, CA, USA) were used for analysis and FACSAria™ II (BD Biosciences) was used for sorting. Flow cytometric data were analyzed with FlowJo sofrware (FlowJo, Ashland, Oregon, USA).

### Antigen Presentation

For cell culture, we used DMC7 medium composed of DMEM containing L-glutamine, high glucose, and pyruvate (HyClone, GE Healthcare Life Sciences, IL, USA) supplemented with 7% fetal bovine serum (Avantor Seradigm), non-essential amino acids (GE Healthcare Life Sciences), antibiotic-antimycotics (GE Healthcare Life Sciences), and 57.2 μM β-mercaptoethanol (Sigma-Aldrich) (31).

**Figure 1:**
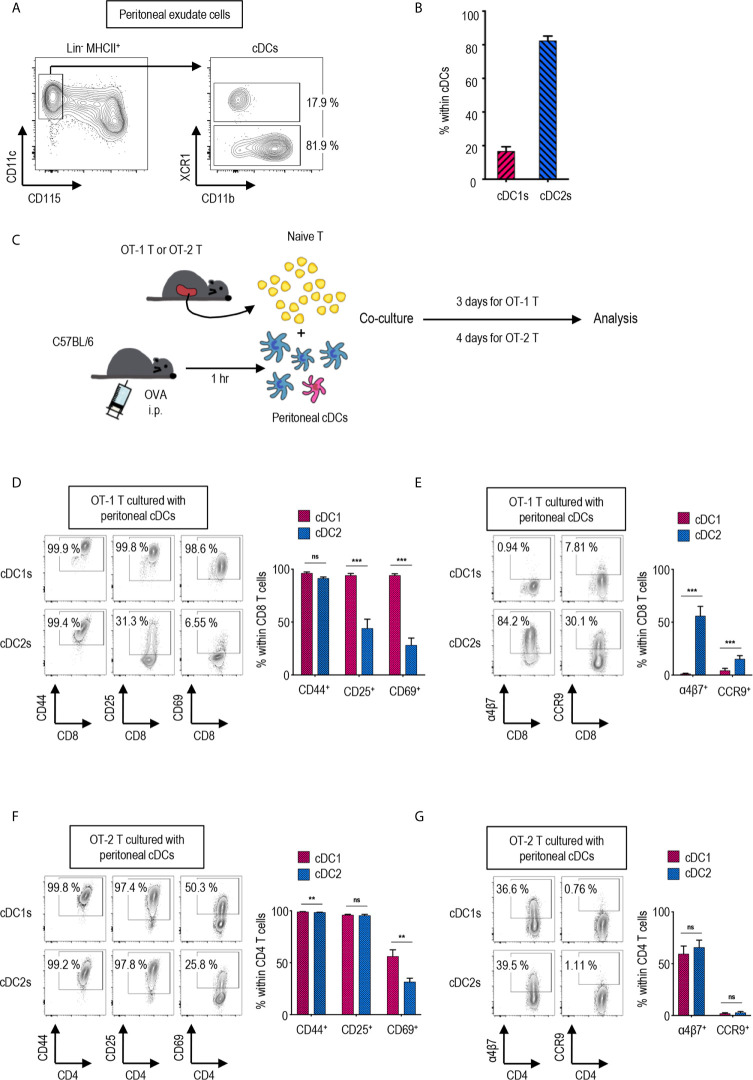
Peritoneal cDC subsets induce distinct T cell responses. **(A)** Gating strategy of cDC subsets in the peritoneal cavity. Cells expressing Lin markers (CD3, CD19 and Ly6G) were excluded and cDCs were gated from MHCII^+^ cells. The percentages of each subset among cDCs are shown (representative of 4 independent experiments). **(B)** The proportion of cDC subsets in the peritoneal cavity. The percentages of each subset among cDCs are shown in bar graph. Data are pooled from 4 independent experiments (n = 4). Error bars indicate mean ± SEM. **(C)** Experimental outline for CD8 **(D, E)** and CD4 **(F, G)** T cell responses. 1.5 mg of Ag (OVA, Grade V) was intraperitoneally (i.p.) injected. An hour later, OVA-laden peritoneal cDC subsets were isolated and co-cultured with either naïve OT-1 (CD8^+^) or OT-2 (CD4^+^) T cells in the ratio of 1:10 (APC:T). OT-1 T cells were analyzed on day 3, and OT-2 T cells on day 4. **(D–G)** CTV^lo^ proliferated T cells are gated and shown. **(D)** Activation status of OT-1 T cells stimulated by peritoneal cDC subsets. Expression of CD44, CD25, and CD69 on OT-1 T cells stimulated by peritoneal cDC subsets are shown. **(E)** Expression of homing molecules, α4β7 and CCR9, on OT-1 T cells stimulated by peritoneal cDC subsets. **(F)** Activation status of OT-2 T cells stimulated by peritoneal cDC subsets. Expression of CD44, CD25, and CD69 on OT-2 cells stimulated by peritoneal cDC subsets are shown. **(G)** Expression of homing molecules, α4β7, and CCR9 on OT-2 T cells stimulated by peritoneal cDC subsets. Data in graphs are pooled from more than 4 **(D, E)** or 2 **(F, G)** independent experiments (n ≥ 5). Error bars indicate mean ± SEM. **p<0.01; ***p<0.001; ns, statistically not significant.

To isolate APCs, mice were injected intraperitoneally (i.p.) or intravenously (i.v.) with soluble OVA (1.5 mg of Grade V, Sigma-Aldrich, unless indicated otherwise), and an hour later, peritoneal exudate cells or splenocytes were harvested as described above. OVA-laden cDC subsets were isolated from MHCII^+^ population, following the gating strategy in **Figure 1A**, excluding cells expressing Lin markers (CD3, CD19, Ly6G, and ICAM2) with the FACSAria™ II cell sorter and used for further experiments.

For naïve T cell isolation, splenic cells from OT-1 and OT-2 mice were enriched by excluding cells expressing CD11b, CD19, CD25, CD44, CD49b, MHCII, F4/80, B220, and CD4 (for OT-1) or CD8 (for OT-2) using biotinylated antibodies and anti-biotin Dynabeads (ThermoFisher Scientific). Purified T cells were labeled with 0.5 mM CFSE or CTV (Thermo Fisher Scientific) according to the manufacturer’s protocol.

For the antigen presentation assay, CFSE or CTV labeled T cells and OVA-laden peritoneal APCs were co-cultured in each well of a 96-well plate. T cells were plated at 25,000 cells, and OVA-laden APCs were mixed with T cells at the ratio of 1:10 unless indicated otherwise. The culture volume was 250 μl. For some experiments, blocking Abs (anti-IL-2Rα, anti-IL-2Rβ, anti-IL-2), retinoic acid, or cytokines (IL-2, IL-6, TGF-β) were treated in written doses. Conditioned medium was made up with 60% of the supernatant from the co-culture of peritoneal cDC subsets and OT-1 T cells and 40% of fresh DMC7 medium.

### Transwell Assay

**Figure 2:**
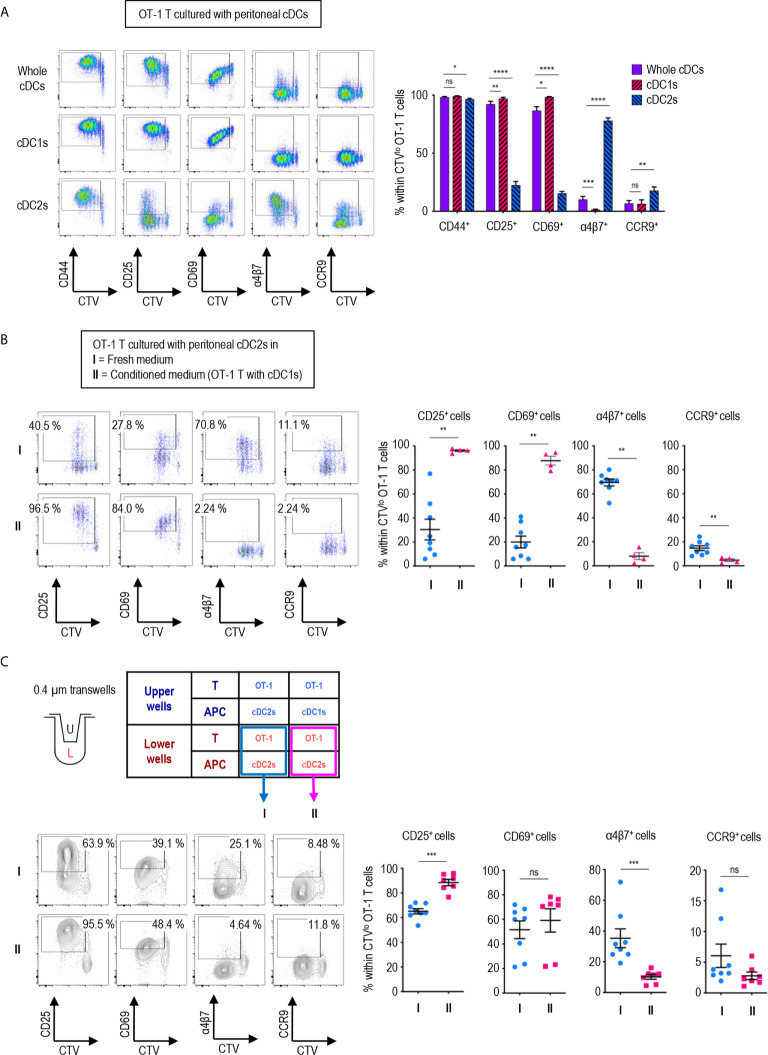
Peritoneal cDC1s play a dominant role in regulating CD8 T cell responses *via* soluble mediators. An hour after injecting 1.5 mg of OVA (Grade V) i.p., OVA-laden peritoneal cDCs were isolated and co-cultured with naïve OT-1 T cells in the ratio of 1:10 (APC:T). OT-1 T cells were analyzed on day 3. **(A)** Expression of CD44, CD25, CD69, α4β7, and CCR9 molecules on OT-1 T cells stimulated by peritoneal whole cDCs and cDC subsets. Live CD8^+^ cells are plotted and the percentages of cells expressing each molecule among CTV^lo^ proliferated OT-1 T cells are shown in a graph. Data are pooled from 7 independent experiments (n ≥ 7). **(B)** OT-1 T cells differentiated by peritoneal cDC2s in conditioned medium. Conditioned medium contained fresh complete medium and the supernatant from co-culture of cDC1s and OT-1 T cells in a 2:3 ratio. OVA-laden peritoneal cDC2s and OT-1 T cells were cultured under either fresh complete medium (I, control) or conditioned medium (II). Live CD8^+^ T cells are plotted and the percentages of cells expressing each surface molecules are shown in graphs. Data are pooled from 2 independent experiments (n ≥ 4). **(C)** OT-1 T cells differentiated in 0.4 µm pore transwells. OT-1 T cells and peritoneal cDCs were co-cultured in transwells for 3 days in combinations as shown in the table. T cells in the lower chamber were analyzed for the expression of CD25, CD69, α4β7, and CCR9. Live CD8^+^ T cells are plotted and the percentages of cells expressing each molecule among CTV^lo^ proliferated OT-1 T cells are shown in graphs. Data are pooled from more than 2 independent experiments (n ≥ 7). Error bars **(A–C)** indicate mean ± SEM. *p<0.05; **p<0.01; ***p<0.001; ****p<0.0001; ns, statistically not significant.

Transwell assays were performed in flat bottom 24-well plates with a pore size of 0.4 μm (SPL, Seoul, Republic of Korea). Each well was filled with either a mixture of OT-1 T cells and OVA-laden peritoneal cDC2s, or a mixture of OT-1 T cells and OVA-laden peritoneal cDC1s as shown (**Figure 2C**). Total reaction volume was 350 μl, and to make cells gather together, the plate was tilted during the co-culture. After 3 days of co-culture, upper chambers were removed and T cells from the lower chambers were analyzed.

### Antibody Array

OT-1 T cells and peritoneal cDC subsets were co-cultured for 3 days as described above. The supernatant of each co-culture sample was collected and the expression of 308 proteins were measured using Mouse L308 Array (Ray Biotech, Norcross, GA, USA). The medium from OT-1 T cells cultured without any APCs was also collected and analyzed to exclude the contents included in the culture medium. The antibody array procedures were performed by e-Biogen (Seoul, Republic of Korea). Global normalization was applied using entire spot intensity.

### ELISA

OT-1 T cells and OVA-laden peritoneal cDC subsets were co-cultured for 3 days and the supernatant was collected. Then, IL-2 was measured from 100 μl of 1/10 diluted supernatant by ELISA following the protocols provided by the manufacturer (BioLegend).

### RNA Processing and RNA-Sequencing Analysis

OT-1 or OT-2 T cells were co-cultured with OVA-laden peritoneal cDC subsets for 3 days as described above. Then, the expanded cells were harvested, and RNA samples were isolated using MiniBEST Universal RNA Extraction kit (TaKaRA Bio, Shiga, Japan). Subsequent mRNA sequencing procedures were performed by Macrogen, Inc (Seoul, Republic of Korea). FPKM values are quantile normalized for comparison. Differential gene expression analysis was performed with ExDega (e-Biogen). Genes with a normalized value of ≤1 in all samples were excluded and only |fold change|≥2 were selected for differentially expressed gene analysis. Pathway analysis was performed using GSEA (Broad Institute). Protein-protein association network was performed with String-DB (http://string-db.org/). RNA-Sequencing data are available from GEO under accession number GSE150863. RNA-Sequencing data of peritoneal cDC subsets (GEO accession number GSE130424) were generated in our previous publication (27), and here analyzed similarly as above.

### Adoptive Cell Transfer and Homing Index Analysis

OT-1 T cells and peritoneal cDC subsets were co-cultured for 3 days as described above. OT-1 T cells stimulated by cDC2s were labeled with CFSE. OT-1 T cells stimulated by either cDC1s or cDC2s with IL-2 (100 ng/ml) were labeled with CTV. 250,000 CFSE-labeled cDC2-stimulated OT-1 T cells were mixed with 250,000 CTV-labeled cDC1-stimulated OT-1 T cells and adoptively transferred i.v. into C57BL/6J recipient mice. For OT-1 T cells stimulated with cDC2s and cDC2s with IL-2, 500,000 OT-1 T cells from each stimulation were mixed and adoptively transferred i.v. into C57BL/6J recipient mice. After 3 hours of adoptive transfer, CFSE-labeled vs. CTV-labeled OT-1 T cells were detected from individual tissues, and the respective homing index (HI) was calculated as [CTV^+^/CFSE^+^]_tissue_/[CTV^+^/CFSE^+^]_input_ (32).

### Statistics

GraphPad Prism 7 and 8 (GraphPad Software, La Jolla, CA) were used for statistical analysis. Statistical comparisons were carried out by using the nonparametric Mann–Whitney tests unless described otherwise. Results are expressed as means ± sem. p<0.05 (*), p<0.01 (**), p<0.001 (***), and p<0.0001 (****) were considered to be statistically significant.

## Results

### Peritoneal cDC1s and cDC2s Induce a Distinct Expression of Activation and Gut-Homing Markers on CD8 T Cells

Peritoneal cDCs can be divided into two subsets depending on the expression of surface marker XCR1 (27). cDCs are identified as lineage marker (Lin)^-^CD115^-^CD11c^+^MHCII^+^ in mouse peritoneal exudate cells (PECs) (**Figure 1A**). Peritoneal cDCs are composed of ca. 20% XCR1^+^ cDC1s and ca. 80% XCR1^-^ cDC2s (**Figure 1B**). To investigate the differences between peritoneal cDC1s and cDC2s in regulation of T cell responses, the expression of activation and gut-homing markers on CD4 and CD8 T cells were evaluated following stimulation with the respective cDC subsets. Prior to isolating the cDC subsets by flow cytometry of PECs, ovalbumin (OVA, grade V, Sigma-Aldrich) was injected i.p. for 1 hour; then, the OVA-laden peritoneal cDC subsets were co-cultured with OVA-specific TCR transgenic CD8^+^ OT-1 or CD4^+^ OT-2 T cells, respectively, in the APC:T ratio of 1:10 (**Figure 1C**). Both cDC1s and cDC2s were able to induce the robust proliferation of naïve OT-1 and OT-2 T cells (**Supplementary Figures 1**, **2A**). However, the expression levels of activation markers (**Figure 1D** and **Supplementary Figure 2B**) and gut-homing markers (**Figure 1E** and **Supplementary Figure 2B**) on the proliferated (CTV^lo^) CD8^+^ OT-1 T cells were significantly different between the stimulations with cDC1s and cDC2s. OT-1 T cells cultured with cDC1s displayed high levels of activation markers, CD25 and CD69, and low levels of gut-homing markers, integrin α4β7 and CCR9, whereas those cultured with cDC2s exhibited contrary patterns. Interestingly, on the surface of CTV^lo^ CD4^+^ OT-2 T cells, those activation (**Figure 1F** and **Supplementary Figure 2C**) and gut-homing markers (**Figure 1G** and **Supplementary Figure 2C**) were expressed similarly in response to both cDC1s and cDC2s. We compared the level of H-2K^b^ molecules presenting OT-1 peptide on peritoneal cDC subsets after i.p. treatment with whole OVA protein. Although the difference was small, the fractions of cDC1s expressing OT-1 peptide bound to H-2K^b^ on surface were higher than those of cDC2s (**Supplementary Figure 3A**). In contrast, when OT-1 peptide was injected i.p., the fractions of cDC1s expressing OT-1 peptide bound to H-2K^b^ on surface were much lower than those of cDC2s (**Supplementary Figure 3B**). These results confirm that peritoneal cDC1s are better at cross-presenting whole protein than peritoneal cDC2s. In addition, both OT-1 T cells stimulated by peritoneal cDC1s or cDC2s pulsed i.p. with OT-1 peptide exhibited the phenotype of OT-1 T cells stimulated by cDC2s pulsed i.p. with OVA protein (**Supplementary Figure 4**). These results suggest that, besides the level of antigen peptide on surface, peritoneal cDC1s are distinct from cDC2s in their antigen-presenting functions. Meanwhile, OT-1 T cells stimulated by OVA-laden splenic cDC1s and cDC2s exhibited the expression of surface markers similarly (**Supplementary Figure 5**) as OT-1 T cells stimulated by peritoneal cDC2s, also implying that peritoneal cDC1s might have distinct functions.

It has been reported that grade V OVA contains a low but detectable level of endotoxin (ca. 0.25 EU/μg) (33, 34). Therefore, by injecting endotoxin-free OVA i.p., we examined the role of endotoxin in the differential expression of markers on CD8^+^ OT-1 T cells between the responses to cDC1s and cDC2s. When laden i.p. with endotoxin-free OVA, both peritoneal cDC1s and cDC2s stimulated the robust proliferation of OT-1 T cells but did not produce the differential expression of markers thereon (**Supplementary Figure 6A**). It was evident that the expression patterns of activation and gut-homing markers on OT-1 T cells stimulated by both cDC subsets laden with endotoxin-free OVA were quite similar to those stimulated by cDC2s laden with grade V OVA (i.e., low levels of activation markers and high levels of gut-homing markers). Meanwhile, when 50 ng of lipopolysaccharide (LPS), the equivalent amount of endotoxin in grade V OVA, was injected i.p. along with endotoxin-free OVA, OT-1 T cells cultured with cDC1s increased the expression of activation markers and decreased the expression of gut-homing markers but those cultured with cDC2s did not change markedly (**Supplementary Figure 6B**). These findings suggest that peritoneal cDC1s are more sensitive to the treatment with low doses of LPS and thus better at activating OT-1 T cells. We examined the levels of co-stimulatory molecules on peritoneal cDC subsets after i.p. injection of PBS or LPS. The expression of CD86 was much higher on cDC1s than on cDC2s, but other co-stimulatory molecules were expressed higher on cDC2s than on cDC1s (**Supplementary Figure 7A**). Also, the expression level of CD86 was prominently upregulated on cDC1s following i.p. treatment with LPS (**Supplementary Figure 7B**). Then, the gene expression profiles of peritoneal cDC subsets were analyzed from the mRNA-sequencing data of peritoneal myeloid mononuclear cells in our previous study (27), which indicates that TLR4 is expressed similarly between peritoneal cDC1s and cDC2s, but the TLR4 signaling molecules are expressed differentially (**Supplementary Figures 8A, B**). Collectively, we conclude that peritoneal cDC1s possess a unique ability to differentially regulate the expression of various markers on CD8^+^ OT-1 T cells in a manner dependent on the presence of endotoxin. Meanwhile, TGF-β, which regulates the expression of homing receptors such as integrins and CD69 (35), and many of the TGF-β signaling molecules are expressed higher in peritoneal cDC2s than in cDC1s (**Supplementary Figure S8C**).

### Expression of Activation and Gut-Homing Markers on CD8 T Cells Are Dominantly Regulated by Peritoneal cDC1s *via* Soluble Mediator(s)

We also compared the expression of activation and gut-homing markers between CD8^+^ OT-1 T cells cultured with peritoneal cDC1s, cDC2, and unseparated whole cDCs. To our surprise, the expression patterns induced by whole cDCs, comprising less than 20% of cDC1s (**Figures 1A, B**), were quite identical to those induced by cDC1s, but not by cDC2s (**Figure 2A**). Meanwhile, the expression levels of those markers on CD4^+^ OT-2 T cells showed no differences between the stimulations with peritoneal cDC1s, cDC2, and whole cDCs (**Supplementary Figure 9**). Since cDC1s, the smaller of the two cDC subsets, seems to dominantly influence the expression of those markers on OT-1 T cells, we scrutinized whether it was involved with soluble mediator(s) emanating from the co-culture of cDC1s and OT-1 T cells. To test this, we collected supernatants from the culture of OT-1 T cells with peritoneal cDC1s or cDC2s, and used the conditioned medium containing each supernatant to culture OT-1 T cells with a respective subset of cDCs. When the conditioned medium from the culture of OT-1 T cells with cDC1s was used, the expression patterns of markers on OT-1 T cells stimulated by cDC2s became similar to those stimulated by cDC1s (**Figure 2B**). However, the conditioned medium from the culture of OT-1 T cells with cDC2s did not affect the expression of markers on OT-1 T cells stimulated by cDC1s (**Supplementary Figure 10**). Therefore, cDC1s play a dominant role among whole peritoneal cDCs in regulating the expression of markers on OT-1 T cells *via* soluble mediator(s). The effect of soluble mediator(s) was further examined using a 0.4 μm transwell co-culture system. When co-cultured with OT-1 T cells and cDC1s in the upper well of the transwell, the OT-1 T cells with cDC2s in the lower well exhibited increase in the expression of CD25 and decrease in the expression of α4β7, as compared to those co-cultured with OT-1 T cells and cDC2s in the upper well (**Figure 2C**). However, the changes of CD69 and CCR9 expression on OT-1 T cells in the lower wells were not significant, suggesting that the soluble mediator(s) emanating from the OT-1 T cells cultured with cDC1s was likely less abundant in the lower well of the transwell assay than in the conditioned medium comprising culture supernatants (**Figure 2C**).

### IL-2 Emanating From CD8 T Cells Regulates T Cell Responses

**Figure 3:**
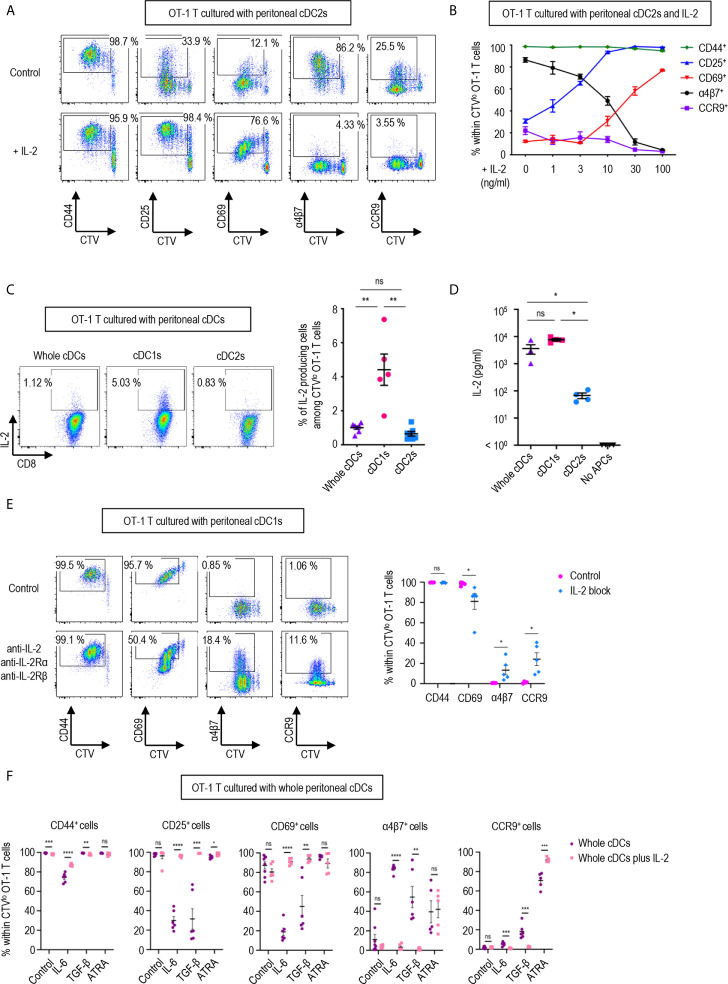
IL-2 produced by CD8 T cells regulates T cell responses. 1.5 mg of OVA (Grade V) was injected into the peritoneal cavity for an hour. OVA-laden peritoneal cDC subsets were sorted and co-cultured with naïve OT-1 T cells for 3 days before analysis. **(A)** Expression of CD44, CD25, CD69, α4β7, and CCR9 molecules on OT-1 T cells stimulated by peritoneal cDC2s with or without 100 ng/ml of IL-2 for 3 days. Live CTV^lo^ CD8^+^ T cells are plotted and the percentages of cells expressing each molecule among CTV^lo^ proliferated OT-1 T cells are denoted (representative of more than 5 independent experiments). **(B)** Effect of graded doses of IL-2 on OT-1 T cells. OT-1 T cells were co-cultured with peritoneal cDC2s with the indicated doses of IL-2 for 3 days. The percentages of cells expressing each molecule among CTV^lo^ proliferated OT-1 T cells are shown in a graph. Data are pooled from more than 3 independent experiments (n = 3). **(C)** Intracellular staining of IL-2. OT-1 T cells were stimulated by peritoneal cDCs for 3 days, and treated with Brefeldin A for the last 4 hours. Live CTV^lo^ CD8^+^ cells are plotted and the percentages of OT-1 T cells expressing IL-2 among CTV^lo^ proliferated OT-1 cells are shown in a graph. Data are pooled from 2 independent experiments (n = 5). **(D)** IL-2 was measured by ELISA from the supernatant collected from OT-1 T cells stimulated by peritoneal cDCs for 3 days (n = 4). **(E)** Expression of CD44, CD69, α4β7, and CCR9 molecules on OT-1 T cells stimulated by peritoneal cDC1s while blocking IL-2 signaling. OT-1 T cells and peritoneal cDC1s were co-cultured with antibodies blocking IL-2 signaling (2 μg/ml of polyclonal anti-IL-2, 10 μg/ml of PC61 anti-IL-2Rα, and 10 μg/ml of TmB1 anti-IL-2Rβ) for 3 days. Live CTV^lo^ CD8^+^ T cells are plotted and the percentages of cells expressing each molecule among CTV^lo^ proliferated OT-1 T cells are shown in a graph. Data are pooled from 3 independent experiments (n = 5). **(F)** Effect of IL-2 and other immunomodulating molecules. OT-1 T cells and peritoneal whole cDCs were co-cultured with or without 1 µg/ml of IL-2 under specified conditions (20 ng/ml of IL-6, 6 ng/ml of TGF-β, or 20 µM of ATRA). The percentages of cells expressing each molecule among CTV^lo^ proliferated OT-1 T cells are shown in graphs. Data are pooled from 2 independent experiments (n ≥ 5). Error bars **(B–F)** indicate mean ± SEM. *p<0.05; **p<0.01; ***p<0.001; ****p<0.0001; ns, statistically not significant.

To identify the soluble mediator(s) that regulates the gene expression, we compared the cytokine expression profiles between the culture supernatants of CD8^+^ OT-1 T cells stimulated by peritoneal cDC1s, cDC2s, and whole cDCs. After screening with antibody microarray for 308 proteins, 33 proteins were detected 1.2-fold or higher in the supernatants from OT-1 T cells cultured with whole cDCs or cDC1s when compared to those from OT-1 T cells cultured with cDC2s (**Table 1**). Then, among these 33 proteins, we have purchased 22 commercially available recombinant proteins and evaluated their effect on the expression of markers on OT-1 T cells cultured with peritoneal cDC2s. The results revealed that only IL-2 was able to modify the expressions of α4β7, CCR9, CD25, and CD69 (**Table 1** and **Figure 3A**), quite as similarly as cDC1s, whole cDCs, and the conditioned media thereof (**Figures 2A, B**). Among these 22 tested proteins, other than IL-2, IL-3 could modulate the expression of α4β7, but not the other markers, on OT-1 T cells. However, no significant change in the expression of markers on OT-1 T cells was observed by blocking the IL-3 signal in the culture with whole cDCs (data not shown).

**Table 1:** List of proteins differentially secreted by OT-1 T cells cultured with different peritoneal cDCs.

Protein	Whole cDCs /cDC2s	cDC1s/cDC2s	Decreased α4β7 on OT-1 T with cDC2s	Decreased CCR9 on OT-1 T with cDC2s	Increased CD25 on OT-1 T with cDC2s	Increased CD69 on OT-1 T with cDC2s
Fas Ligand	1.4	1.9	No	No	No	No
MMP-3	1.3	1.9	No	No	No	No
Activin A	1.7	1.8	No	No	No	No
DAN	1.3	1.7	No	No	No	No
IL-20	1.3	1.6	No	No	No	No
TGF-b1	1.6	1.6	No	No	No	No
IL-17	1.8	1.6	No	No	No	No
IL-2	1.2	1.6	**Yes**	**Yes**	**Yes**	**Yes**
SCF	1.3	1.5	No	No	No	No
TSLP	1.6	1.5	No	No	No	No
Pentraxin3	1.3	1.5	No	No▴	No	No
Osteoprotegerin	1.3	1.5	No	No	No	No
Granzyme G	1.3	1.4	No	No▴	No	No
TECK	1.2	1.4	–	–	–	–
VEGF-D	1.3	1.4	–	–	–	–
Eotaxin-2	1.6	1.4	No	No▴	No	No
L-Selectin	1.2	1.4	–	–	–	–
Kremen-2	1.3	1.3	–	–	–	–
TGFb3	1.2	1.3	No	No	No	No
EG-VEGF	1.2	1.3	–	–	–	–
IFN-g	1.5	1.3	No	No	No	No
TL1A/TNFSF15	1.2	1.3	No	No	No	No
CCL17	1.3	1.3	No	No	No	No
IGFBP-3	1.2	1.3	No	No	No	No
IGFBP-1	1.2	1.2	No	No▴	No	No
Decorin	1.3	1.2	–	–	–	–
Osteoactivin	1.6	1.2	–	–	–	–
IL-6	1.3	1.2	No	No	No	No
IL-3	1.3	1.2	**Yes**	No▴	No	No
IFN-beta	1.3	1.2	–	–	–	–
MMP-14/LEM-2	1.3	1.2	–	–	–	–
Lefty-1	1.3	1.2	–	–	–	–
VEGFC	1.4	1.2	–	–	–	–

1.5 mg of OVA was given i.p. for 1 hour. OVA-laden peritoneal cDC subsets and whole cDCs were isolated and co-cultured with naïve OT-1 T cells in the ratio of 1:10 (APC:T). Each supernatant was collected and analyzed by antibody microarray. The list includes soluble factors secreted 1.2-fold or higher in the supernatants from OT-1 T cells cultured with either cDC1s or whole cDCs, as compared to those cultured with cDC2s. Each protein in the list shows the value of fold change and the result of its influence on OT-1 T cells cultured with cDC2s. ▴indicates the increase of CCR9 instead of the decrease. Change to the indicated phenotype is marked as Yes in bold.

In a dose-dependent manner, IL-2 was able to up-regulate the expression of activation markers CD25 and CD69, and suppressed the levels of integrin α4β7 and CCR9 (**Figure 3B**). We also verified that the expression of IL-2 was prominently elevated in the OT-1 T cells stimulated by peritoneal cDC1s and their culture supernatants (**Figures 3C, D**). Then, we validated the role of IL-2 by blocking the IL-2 signal in OT-1 T cells cultured with peritoneal cDC1s. The effect of each individual antibody of anti-IL-2Rα, anti-IL-2Rβ, or anti-IL-2, including JES6-1 known to efficiently inhibit the response of CD8^+^ T cells to IL-2 *in vitro* (14, 36), was not effective to reverse the phenotype of OT-1 T cells (**Supplementary Figure 11**). With the combination of anti-IL-2Rα, anti-IL2Rβ, and anti-IL-2, the level of CD69 reduced and the expression of α4β7 and CCR9 increased significantly (**Figure 3E** and **Supplementary Figure 11**). We also confirmed the effect of IL-2 on CD4^+^ OT-2 T cells cultured with peritoneal cDC2s where the addition of IL-2 caused the expression of α4β7 to decrease and the expression of CD69 to increase significantly (**Supplementary Figure 12**). Signaling molecules such as retinoic acid, TGF-β, and IL-6 are known to play an essential role in imprinting gut-homing markers and modulating the activation status of T cells (7, 37, 38). We evaluated whether IL-2 could influence the effect of those molecules in OT-1 T cells cultured with peritoneal cDCs. The addition of IL-6 or TGF-β into OT-1 T cells cultured with whole cDCs down-regulated the expression of CD25 and CD69, and up-regulated the level of α4β7; besides, IL-6 down-regulated CD44 expression and TGF-β up-regulated CCR9 expression, respectively (**Figure 3F**). On the other hand, the addition of retinoic acid only up-regulated the expression of CCR9 (**Figure 3F**). However, when IL-2 was included in the cultures, the effects of IL-6 and TGF-β were all reverted but not those of retinoic acid (**Figure 3F**). In the meantime, the dominant control of IL-2 over the effect of IL-6 and TGF-β was also demonstrated in the culture of OT-1 T cells with peritoneal cDC2s, where the reverting effect of IL-2 over retinoic acid was limited to the expression of α4β7 (**Supplementary Figure 13**). Therefore, IL-2 emanating from OT-1 T cells stimulated by peritoneal cDC1s can act in a dominant manner to regulate the expressions of activation and gut-homing markers on T cells.

### Peritoneal cDC Subsets Differentially Regulate the Gene Expression Profiles of T Cells

**Figure 4:**
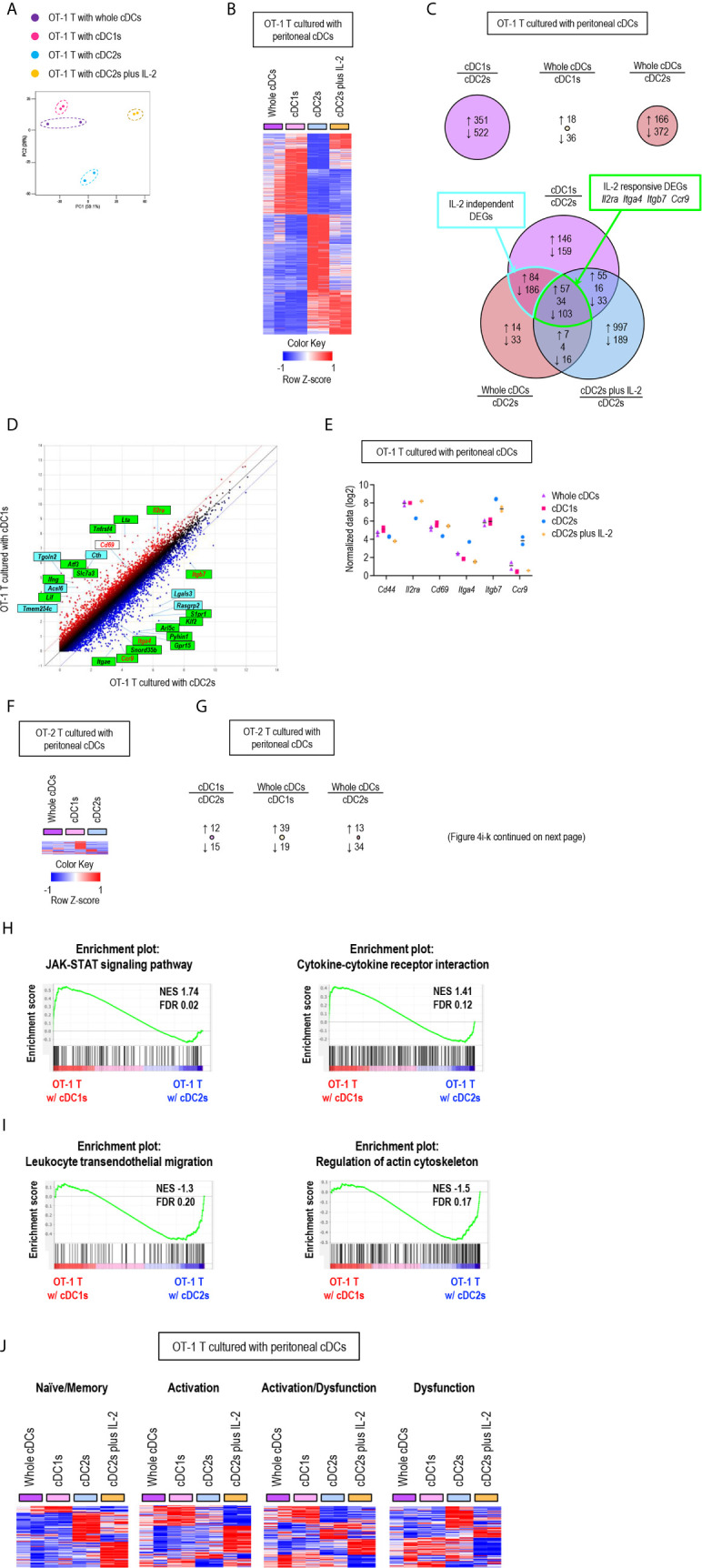
Gene expression profiles of T cells are differentially regulated by peritoneal cDC subsets. 1.5 mg of OVA (Grade V) was injected into the peritoneal cavity for an hour. OVA-laden peritoneal cDC subsets were isolated and co-cultured with either naïve OT-1 or OT-2 T cells for 3 days before performing mRNA-sequencing for the transcriptional profiling analyses. Transcriptional profiling analyses were also performed for OT-1 T cells co-cultured with OVA-laden peritoneal cDC2s in 100 ng/ml of IL-2 for 3 days. **(A)** PCA analysis of OT-1 T cells stimulated by peritoneal cDCs. **(B)** Heatmap of the 873 differentially expressed genes (log2 fold change >1) between OT-1 T cells stimulated by peritoneal cDC1s and cDC2s. Two replicates per subset are shown. Color scale depicts z-score. **(C)** Venn diagram shows the overlap between genes with fold change > 2 in OT-1 T cells stimulated by peritoneal cDCs. Number of genes that are increased (up-arrow, red), decreased (down-arrow, blue), or contra regulated (no-arrow, black) are shown. **(D)** Scatter plot of log intensity comparing gene expression between OT-1 T cells stimulated by peritoneal cDC1s and cDC2s. Top 10 genes differentially expressed (black) and *Il2ra*, *Cd69*, *Itga4*, *Itgb7*, and *Ccr9* (red) are shown. The genes within green boxes are IL-2 responsive and blue boxes are IL-2 independent. **(E)** Expression (normalized value, log2) of *Cd44*, *Il2ra*, *Cd69*, *Itga4*, *Itgb7*, and *Ccr9* in OT-1 T cells stimulated by peritoneal cDCs (n = 2). **(F)** Heatmap of the 58 differentially expressed genes (log2 fold change >1) between OT-2 T cells stimulated by peritoneal cDC1s and cDC2s. Two replicates per subset are shown. Color scale depicts z-score. **(G)** Number of differentially expressed genes (log2 fold change >1) that are increased (up-arrow, red), or decreased (down-arrow, blue) in OT-2 T cells stimulated by peritoneal cDCs are shown. **(H, I)** Enriched pathway analysis using the C7 GSEA collection (ImmunoSigDB). **(H)** Representative enrichment plot from OT-1 T cells stimulated by peritoneal cDC1s compared to cDC2s, “JAK_STAT_signaling_pathway” and “Cytokine_cytokine_receptor_interaction”. **(I)** Representative enrichment plot from OT-1 T cells stimulated by peritoneal cDC2s compared to cDC1s, “Leukocyte_transendothelial_migration” and “Regulation_of_actin_cytoskeleton”. **(J)** Heatmap of the genes involved in CD8 T cell status.

To further characterize the regulatory role of peritoneal cDCs over T cell responses, we performed global mRNA sequencing. CD8^+^ OT-1 T cells were cultured with either whole or individual subsets of OVA-laden peritoneal cDCs in the APC:T ratio of 1:10 for 3 days. OT-1 T cells were also cultured with OVA-laden peritoneal cDC2s in the medium containing IL-2 (cDC2s/IL-2). Then, the gene expression profiles were analyzed and compared between the cells in each co-culture, where almost all cells were expanded T cells (more than 99%) but very few were cDCs (less than 1%). Similarities and differences between the transcriptomes of T cells cultured with peritoneal cDCs were analyzed by principal component analysis (PCA) (**Figure 4A**). The gene expression profiles of OT-1 T cells cultured with cDC1s clustered remotely from those cultured with cDC2s, indicating that the expression of genes in T cells are differentially regulated by different peritoneal cDC subsets. Meanwhile, the gene expression profiles of OT-1 T cells cultured with peritoneal whole cDCs clustered very closely to those cultured with peritoneal cDC1s but apart from those cultured with peritoneal cDC2s, suggesting the superior influence of cDC1s, compared to cDC2s, over the gene expression of T cells. We also investigated the impact of IL-2 on the gene expression profile of OT-1 T cells cultured with peritoneal cDC2s. In PCA, by the addition of IL-2, OT-1 T cells cultured with peritoneal cDC2s clustered distinctively from those cultured with peritoneal cDC subsets without IL-2 (**Figure 4A**). It is notable that the first principal component (PC1) distinguishes the OT-1 T cells stimulated by cDC2s with IL-2 from the other clusters of those stimulated by cDC subsets without IL-2. However, the PC2 discriminates the OT-1 T cells stimulated by cDC2s with IL-2, clearly from those stimulated by cDC2s without IL-2, but not from those stimulated by cDC1s or whole cDCs without IL-2 (**Figure 4A**). These results indicate that there exist IL-2 dependent and IL-2 independent sets of genes expressed differentially between the OT-1 T cells cultured with cDC1s versus cDC2s.

Then, we examined the differentially expressed genes (DEGs) between OT-1 T cells cultured with cDC1s versus cDC2s (**Figure 4B**), where 351 genes were up-regulated in the OT-1 T cells cultured with cDC1s and 522 genes were up-regulated in those cultured with cDC2s (**Figure 4C**, upper left diagram). We also compared the DEGs between OT-1 T cells cultured with whole cDCs versus cDC1s, where only 54 genes were differentially expressed (**Figure 4C**, upper middle diagram), and those between OT-1 T cells cultured with whole cDCs versus cDC2s, where 538 genes were differentially regulated (**Figure 4C**, upper right diagram). Notably, more than 80% of the 538 DEGs between the OT-1 T cells cultured with whole cDCs versus cDC2s were overlapped with the DEGs between those cultured with cDC1s versus cDC2s (**Figure 4C**, lower diagram), also suggesting the dominant role of cDC1s in controlling the gene expression of T cells. We extended the DEG analysis to include the OT-1 T cells stimulated by cDC2s with IL-2. Among the 464 DEGs common to both gene expression profiles of OT-1 T cells stimulated by whole cDCs and cDC1s over those stimulated by cDC2s, 160 genes were overlapped with the DEGs in those stimulated by cDC2s with IL-2 over those stimulated by cDC2s without IL-2 (**Figure 4C**, lower diagram). These overlapping genes, such as *Il2ra*, *Itga4*, *Itgb*, and *ccr9*, were classified as IL-2 responsive DEGs, and non-overlapping genes as IL-2 independent DEGs (**Figures 4C–E**). It is notable that, while the expression of many genes involving T cell activation and differentiation in OT-1 T cells was distinctively affected by the treatment of IL-2 during stimulation with peritoneal cDC2s, the gene expression of IL-2 and CD62L was not influenced by the treatment of IL-2 (**Supplementary Figure 14**). Meanwhile, we also analyzed the gene expression profiles of OT-2 T cells stimulated by different subsets of peritoneal cDCs. Unlike CD8^+^ OT-1 T cells, there existed only a few DEGs between CD4^+^ OT-2 T cells cultured with different peritoneal cDCs (**Figures 4F, G**).

Next, we conducted gene set enrichment analysis (GSEA) to identify pathways and processes associated with the differential responses of OT-1 T cells to different cDC subsets. OT-1 T cells cultured with cDC1s were enriched with genes involving the pathways for JAK-STAT signaling and cytokine-cytokine receptor interaction (**Figure 4H**). Meanwhile, those cultured with cDC2s were enriched with genes in the pathways involving leukocyte transendothelial migration and actin cytoskeleton regulation (**Figure 4I**). Because the numbers of genes were not large enough for GSEA, we then analyzed the pathways involved in IL-2 responsive and IL-2 independent DEGs using StringDB and constructed a protein-protein association network. In the IL-2 responsive gene set, we identified that the up-regulated genes were involved in cytokine-cytokine receptor interaction, such as *Ifng*, *Lta*, *Fasl*, *Tnf*, *Ccl3*, *Ccl4*, and *Ccr4*, whereas the down-regulated genes were involved in leukocyte migration, such as *Itgb7*, *Itga4*, *Itgae*, and *S1pr1* (**Supplementary Figure 15**). In the IL-2 independent gene set, we found that the up-regulated genes were involved in metabolic pathways, such as *Cth*, *Acsl6*, *Bcat1*, *Polr1b*, *Ctps*, *Ak4*, *Gls2*, and *Nos2* (**Supplementary Figure 16**). In addition, we also looked into gene expression modules related to the functional status of CD8^+^ T cells (39). OT-1 T cells, stimulated by peritoneal cDC2s, up-regulated the expression of a considerable number of genes involved in dysfunction but fewer number of genes involved in activation. In contrast, OT-1 T cells, stimulated by whole cDCs, cDC1s, or cDC2s with IL-2, induced a significantly greater number of genes involved in activation than those stimulated by cDC2s. However, the up-regulated genes in OT-1 T cells stimulated by cDC2s with IL-2 were rather different from those in OT-1 T cells stimulated by whole cDCs and cDC1s (**Figure 4J**). Thus, peritoneal cDC2s likely cause the dysfunction of OT-1 T cells, while cDC1s contribute to the activation of OT-1 T cells *via* controlling the expression of a large number of genes in IL-2 dependent as well as IL-2 independent manners. All in all, our analyses reveal the distinctive features of peritoneal cDC1s and cDC2s that regulate the gene expression in CD8^+^ OT-1 T cells.

### Different Subsets of Peritoneal cDCs Induce Distinct Homing Properties of T Cells

**Figure 5:**
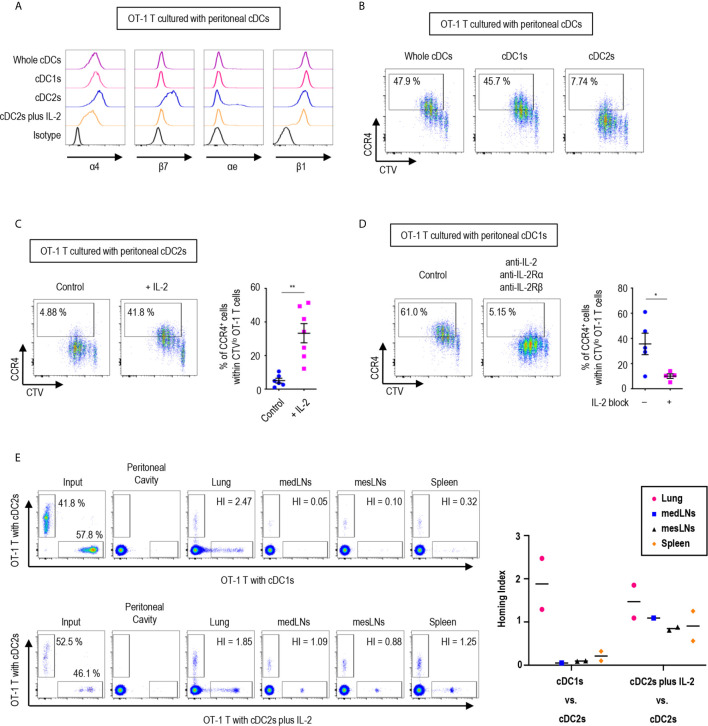
Peritoneal cDCs induce CD8 T cells with distinct homing properties. 1.5 mg of OVA (Grade V) was injected into the peritoneal cavity for an hour. OVA-laden peritoneal cDC subsets were isolated and co-cultured with naïve OT-1 T cells for 3 days before analysis. **(A)** OT-1 T cells stimulated by cDCs were stained for integrins α4, β7, αe, and β1 (representative of more than 3 independent experiments). **(B)** Surface expression of CCR4 in OT-1 T cells stimulated by peritoneal cDCs are shown (representative of 4 independent experiments). **(C)** Effect of IL-2 on CCR4 expression. OT-1 T cells and peritoneal cDC2s were co-cultured with or without 100 ng/ml of IL-2 for 3 days. Live CTV^lo^ CD8^+^ T cells are plotted and the percentages of cells expressing CCR4 among CTV^lo^ proliferated OT-1 T cells are shown in a graph. Data are pooled from 2 independent experiments (n ≥ 6). Error bars indicate mean ± SEM. **(D)** Expression of CCR4 on OT-1 T cells stimulated by peritoneal cDC1s while blocking IL-2 signaling. OT-1 T cells and peritoneal cDC1s were co-cultured with antibodies blocking IL-2 signaling (2 μg/ml of polyclonal anti-IL-2, 10 μg/ml of PC61 anti-IL-2Rα, and 10 μg/ml of TmB1 anti-IL-2Rβ) for 3 days. Live CTV^lo^ CD8^+^ T cells are plotted and the percentages of cells expressing CCR4 among CTV^lo^ proliferated OT-1 T cells are shown in a graph. Data are pooled from 2 independent experiments (n ≥ 4). Error bars indicate mean ± SEM. **(E)** Homing index (HI) measured by competitive adoptive transfer experiments. OT-1 T cells were stimulated by cDC2s (labeled with CFSE), cDC1s (labeled with CTV), or cDC2s plus IL-2 (labeled with CTV). Labeled OT-1 T cells were mixed 1:1 and adoptively transferred intravenously (i.v.) into recipient mice. A total of 500,000 cells containing an equal mixture of OT-1 T cells respectively stimulated by cDC1s vs. cDC2s (upper panels) or a total of 1,000,000 cells containing an equal mixture of OT-1 T cells respectively stimulated by cDC2s plus IL-2 vs. cDC2s (lower panels) were adoptively transferred. After 3 hours of competitive adoptive transfer, HI of each tissue was determined. HI was calculated as [CTV^+^/CFSE^+^]_tissue_/[CTV^+^/CFSE^+^]_input_. Graph shows means of 2 HI values from 2 independent experiments. *p<0.05; **p<0.01.

Since the expression of gut-homing markers, α4β7 and CCR9, on CD8^+^ OT-1 T cells was differentially regulated between the stimulation with peritoneal cDC1s and cDC2s, we further examined the expression of other integrins and chemokine receptors. Integrins are expressed on cell surfaces as heterodimers formed by α and β subunits. An integrin α4 can form heterodimers with either integrin β1 or β7, while integrin β7 can be paired with either integrin α4 or αe (**Supplementary Figure 17**). In particular, the heterodimers formed with integrin β7 play critical roles in gut trafficking of leukocytes (40). Therefore, we examined the expression of individual integrin subunits on OT-1 T cells following stimulation with peritoneal cDCs (**Figure 5A** and **Supplementary Figures 18, 19**). In accordance with their expression of α4β7, both integrin subunits α4 and β7 were expressed on OT-1 T cells stimulated by cDC2s. Meanwhile, OT-1 T cells stimulated by cDC2s with IL-2 sharply down-regulated the expression of integrin β7. Similarly, OT-1 T cells stimulated by whole cDCs and cDC1s failed to express integrin β7 but maintained the expression of integrin α4 on the cell surface. Additional evaluation of integrins αe and β1, the other binding partners of integrins β7 and α4 respectively, revealed that cDC2s up-regulated both integrins αe and β1 on OT-1 T cells, whereas whole cDCs, cDC1s, and cDC2s with IL-2 induced β1 but not αe (**Figure 5A** and **Supplementary Figures 18, 19**). Collectively, the expression of integrins β7 and αe, best known for gut-homing properties, on CD8^+^ OT-1 T cells was sustained by peritoneal cDC2s but suppressed by cDC1s or cDC2s with IL-2.

We also examined the influence of peritoneal cDCs on the expression of chemokine receptor genes in OT-1 T cells (**Supplementary Figure 20**). In addition to CCR9, the stimulation with peritoneal cDC2s strongly induced the gene expression of *Cxcr3, Cxcr4, Cxcr6*, and *Xcr1*, which are homing receptors to multiple organs including both lymphoid and non-lymphoid tissues (41, 42). Peritoneal cDC1s significantly up-regulated the gene expression of *Ccr4, Ccr6*, and *Ccr8*, known to be important in homing to the lung and the skin (32, 41, 42). Meanwhile, OT-1 T cells stimulated by cDC2s with IL-2 increased the gene expression of more diverse chemokine receptors, including *Ccr2, Ccr3, Ccr4, Ccr5, Ccr10, Cxcr3, Cxcr4*, and *Cxcr6* (**Supplementary Figure 20**). Then, we assessed the surface expression of chemokine receptors and confirmed the augmented expression of CCR4 on the surface of OT-1 T cells stimulated by cDC1s as compared to those stimulated by cDC2s (**Figure 5B**). The low expression of CCR4 on OT-1 T cells stimulated by cDC2s was influenced by treatment of the conditioned medium from the culture of OT-1 T cells with cDC1s (**Supplementary Figure 21**). We then demonstrated that the addition of IL-2 to the culture with cDC2s significantly up-regulated the expression of CCR4 on OT-1 T cells (**Figure 5C**), and that the inhibition of IL-2 signaling significantly reduced the expression of CCR4 on OT-1 T cells cultured with cDC1s (**Figure 5D**). Therefore, the level of CCR4 on CD8^+^ OT-1 T cells are up-regulated by the stimulation with peritoneal cDC1s in an IL-2 dependent manner. Meanwhile, the expression of TGF-β and TGF-β signaling molecules, which regulate the expression of homing receptors (35), in OT-1 T cells is not markedly affected by the stimulation with different peritoneal cDC subsets and IL-2 (**Supplementary Figure 22**).

To determine whether OT-1 T cells stimulated by different peritoneal cDC subsets possess distinct properties to migrate into peripheral tissues *in vivo*, we examined their homing capacities using competitive adoptive transfer experiments (32). OT-1 T cells stimulated and expanded by 2 different peritoneal cDC subsets were labeled respectively with 2 different fluorescent dyes (i.e., CFSE and CTV), mixed in an equal ratio, and then adoptively transferred intravenously into recipient mice. At 3 hours following adoptive transfer, various lymphoid and peripheral tissues were harvested and analyzed for the presence of fluorescently labeled OT-1 T cells. The HI (the ratio of [CTV^+^ cells/CFSE^+^ cells] in tissue to [CTV^+^ cells/CFSE^+^ cells] in input) indicated that OT-1 T cells stimulated by cDC2s were able to traffic to various tissues, including the mediastinal lymph nodes (medLNs), mesenteric lymph nodes (mesLNs), spleen, and lungs, while those stimulated by cDC1s migrated preferentially to the lung but poorly to the lymphoid tissues (**Figure 5E**, upper panels). When the migration of OT-1 T cells was compared between the cultures with cDC2s versus cDC2s with IL-2, both groups of OT-1 T cells were able to migrate to various tissues (**Figure 5E**, lower panels). It was notable that none of the adoptively transferred OT-1 T cells were detected in exudates from the peritoneal cavity (**Figure 5E**). In addition, despite the significantly elevated levels of gut-homing molecules on OT-1 T cells stimulated by cDC2s, none of those were detectable in the gut tissues (**Supplementary Figure 23**). These results suggest that OT-1 T cells stimulated by cDC1s express lung-homing molecules, such as CCR4, as the dominant homing mechanism, whereas those stimulated by cDC2s support broad homing properties.

## Discussion

Upon stimulation with APCs, T cells undergo several changes. T cells become activated, differentiate into specific subsets, and alter their tissue-specific homing molecules (41, 43, 44). We evaluated these features of T cells stimulated by peritoneal cDC subsets and found that different cDC subsets made differences in the expression of activation and homing receptors on CD8^+^ OT-1 T cells. Stimulation with cDC1s was superior in activating OT-1 T cells. Also, cDC1s promoted the lung-homing capacity of OT-1 T cells while cDC2s increased the expression of gut-homing molecules on the surface. Like all other tissues, the peritoneal cavity contains a much smaller number of cDC1s than that of cDC2s within the whole cDC population. However, the phenotypic and functional outcomes of whole peritoneal cDCs were quite similar to those of peritoneal cDC1s. Global mRNA-sequencing analysis of transcriptomes also confirmed that the overall gene expression profile of OT-1 T cells stimulated by whole cDCs were similar to those stimulated by cDC1s but not cDC2s. Therefore, our study demonstrates that peritoneal cDC1s play a dominant role over other cDCs in controlling the responses of CD8^+^ OT-1 T cells.

The cytokine milieu is important for the differentiation of T cells. It is shown that TCR-stimulated T cells and bystander T cells are differentially influenced and polarized in the culture containing IL-2 (45). Recent studies on the cytokine milieus that lead the differentiation of T cells into specific subsets indicated that DCs actively take part in generating such conditions. DCs can directly secrete certain cytokines and/or modulate the response to specific cytokines (22, 23). DCs can also indirectly regulate the level of particular cytokines by inducing T cells to secrete and/or to consume those cytokines (21, 22). Besides, CD25 on Ag-presenting DCs can participate in *trans*-presentation of IL-2 to Ag-responding T cells (14, 46). Meanwhile, IL-2 immunotherapy is found to expand and activate the population of DCs, and thus boost immunity (47). Our present study illustrated that peritoneal cDC1s, but not cDC2s, are able to promote OT-1 T cells to secrete a high level of IL-2. Although the increased level of IL-2 modulates the gene expression profile of OT-1 T cells stimulated by cDC2s to mimic that of OT-1 T cells stimulated by cDC1s, a significant number of genes are still regulated by cDC2s independently of IL-2. In fact, IL-2 is a complicated cytokine producing different outcomes depending on the type, strength, and duration of signals (14, 48). For example, a strong IL-2 signal, in combination with inflammatory cytokines, induces the expression of transcription factor T-bet and Blimp-1, which in turn promotes the differentiation of short-lived effector CD8 T cells (20). On the other hand, weak IL-2 signals are involved in the formation of memory T cells (18, 19). Thus, we cannot exclude the possibility that DEGs between the stimulations with cDC1s and cDC2s with IL-2 may be due to the inadequate dose and/or the prolonged duration of IL-2 signals. In addition, other modulating factors derived from cDC1s may act on T cells synergistically and/or cooperatively with IL-2, which requires further studies.

We also discovered that peritoneal cDC1s and whole cDCs induce OT-1 T cells to up-regulate CCR4, a chemokine receptor for lung and inflamed tissues (41, 42), and to down-regulate α4β7 and CCR9, homing molecules for gut tissues (49, 50), in an IL-2 dependent manner. On the other hand, peritoneal cDC2s induce OT-1 T cells to express a variety of chemokine receptors and integrins, homing molecules for various tissues, as well as CD62L, a homing receptor for lymphoid tissues. Consistent with this observation, in competitive adoptive transfer assays, most of the OT-1 T cells activated by cDC1s migrate into the lung, whereas those stimulated by cDC2s migrate into various lymphoid tissues as well as the lung. In the meantime, T cells activated by cDC2s with IL-2 show the homing properties of T cells induced by both peritoneal cDC1s and cDC2s. They down-regulate gut-homing molecules but up-regulate both CCR4 and various other chemokine receptors expressed by OT-1 T cells stimulated by cDC2s. Accordingly, their homing properties are not limited to the lung but also able to migrate into various lymphoid tissues. Notably, despite the augmented expression of gut-homing molecules, OT-1 T cells stimulated by cDC2s are unable to migrate into gut tissues in competitive adoptive transfer assays. Besides these findings, no significant gut-homing properties of OT-1 T cells are observed following adoptive transfer into the recipient mice treated with OVA and IL-2 (data not shown).

The mechanisms of how different peritoneal cDC subsets lead to the different outcomes of CD8^+^ T cell responses are not fully recognized *in vivo*. It is notable that, although the expression of TGF-β in OT-1 T cells is not influenced by the stimulation with different peritoneal cDC subsets, TGF-β is expressed higher in peritoneal cDC2s than in cDC1s. Since TGF-β mediates complex effects on the immune system by dampening or promoting T cell responses as well as regulating the expression of integrins and CD69 (35, 38, 51), the different expression of TGF-β between peritoneal cDC subsets might also be involved in controlling the differential expression of genes in CD8^+^ T cells. Interestingly, the dominant feature of cDC1s on controlling the responses of Ag-responding CD8^+^ T cells is only observed when a small amount of LPS is co-delivered i.p. with the Ag, indicating that peritoneal cDC1s are better at activating OT-1 T cells, in part, because they are more sensitive to LPS. Although the expression of TLR4 gene in both peritoneal cDC1s and cDC2s is similar, the signaling molecules are expressed differently. Also, the higher expression and induction of CD86 on peritoneal cDC1s are notable, while other co-stimulatory molecules are expressed higher on peritoneal cDC2s with/without LPS treatment. Meanwhile, peritoneal cDC subsets did not induce distinct CD8 T cell responses nor showed dominance over each other when Ag and LPS was co-delivered *in vitro* (data not shown). This likely suggests that, to acquire the dominant feature, cDC1s need maturation stimuli directly as well as indirectly from the *in vivo* treatment of LPS. It will be important to elucidate the nature of stimulating signals inside the peritoneal cavity that maturate cDC1s and the underlying mechanisms of how different cDC subsets lead to distinct T cell responses.

## Data Availability Statement

The datasets presented in this study can be found in online repositories. The names of the repository/repositories and accession number(s) can be found in the article/**Supplementary Material**.

## Ethics Statement

The animal study was reviewed and approved by Institutional Animal Care and Use Committees of the Yonsei University College of Medicine.

## Author Contributions

Conceptualization: CP. Methodology: MS, HN, and CP. Formal analysis, MS and HN. Investigation: MS, HN, HS, SR, SP, HI, WC, JP, and SH. Writing – original draft: MS, HN, and CP. Writing – review & editing: MS and CP. Visualization: MS and HN. Supervision: CP. Project administration: MS and HN. Funding acquisition: HN, MC, and CP. All authors contributed to the article and approved the submitted version.

## Funding

We were supported by grants from the National Research Foundation of Korea to CP (2017M3A9C8064887, 2019R1F1A1041700), HYN (2017R1A6A3A11028388), and MC (2019R1F1A1053841), the “Dongwha” Faculty Research Assistance Program of the Yonsei University College of Medicine to CP (6-2020-0111), and the Brain Korea 21 PLUS/FOUR Project for Medical Science, Yonsei University. 

## Conflict of Interest

CP is employed by GENUV Inc.

The remaining authors declare that the research was conducted in the absence of any commercial or financial relationships that could be construed as a potential conflict of interest.

## Abbreviations

Ags, antigens; APCs, antigen presenting cells; cDC, classical dendritic cells; DCs, dendritic cells; DEGs, differentially expressed genes; GSEA, gene set enrichment analysis; IEC, intestinal epithelial cell; i.p., intraperitoneal; i.v., intravenous; Lin, lineage; LPL, lamina propria lymphocytes; LPS, lipopolysaccharide; medLNs, mediastinal lymph nodes; mesLNs, mesenteric lymph nodes; OVA, ovalbumin; PBS, phosphate buffered saline; PCA, principal component analysis; RBCs, red blood cells; Tc, CD8 cytotoxic T cell.
