# Supplementary material for: Global Gene Expression of T Cells Is Differentially Regulated by Peritoneal Dendritic Cell Subsets in an IL-2 Dependent Manner
Source: Front Immunol. 2021 May 17;12:648348. doi: 10.3389/fimmu.2021.648348 (PMC8165281; doi:10.3389/fimmu.2021.648348)
Supplement: Supplementary file 1 [file Presentation_1.pptx]

## Slide 1
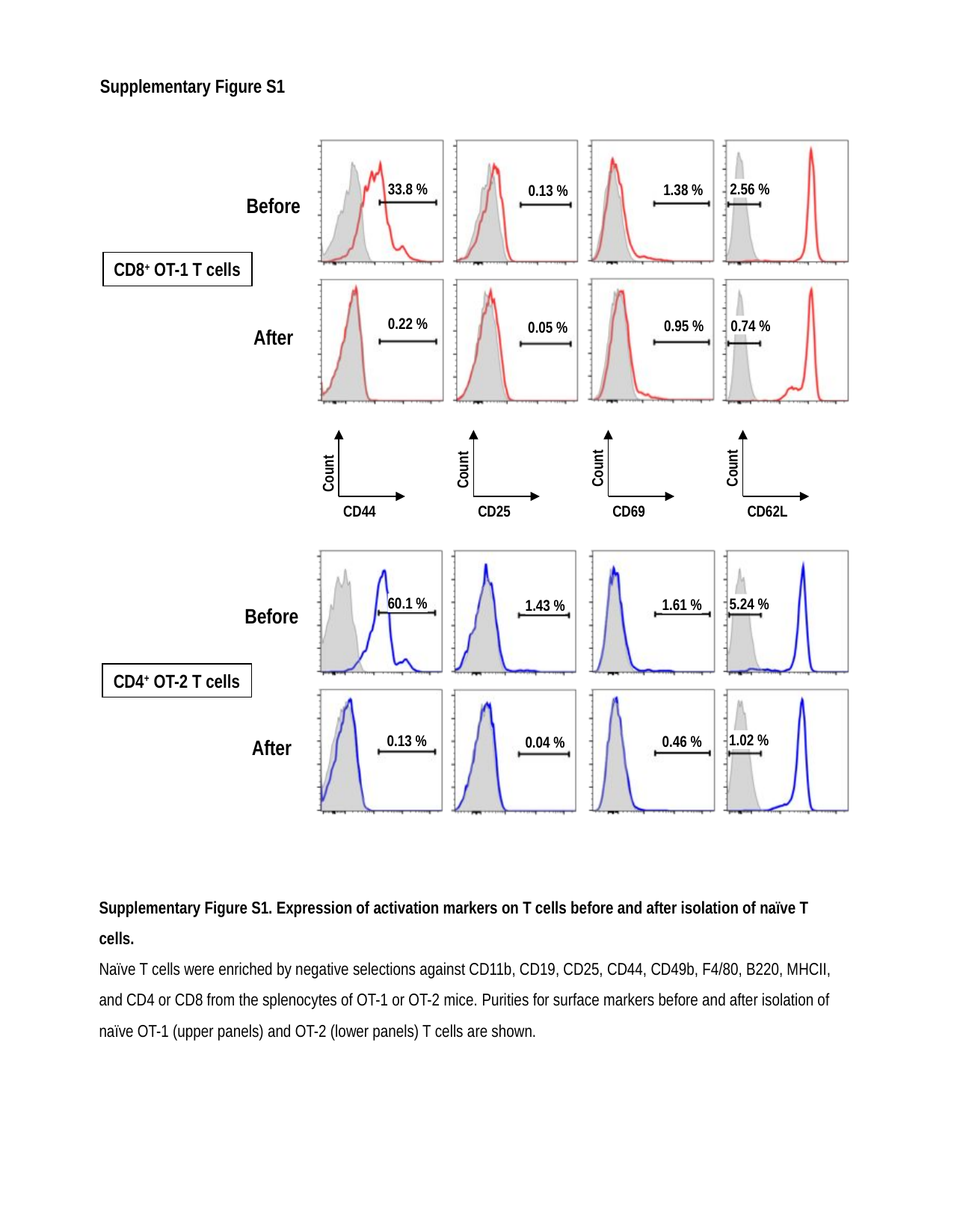

Supplementary Figure S1
33.8 %
2.56 %
1.38 %
0.13 %
Before
0.22 %
0.74 %
0.95 %
0.05 %
After
CD8+ OT-1 T cells
Count
CD44
Count
CD25
Count
CD69
Count
CD62L
60.1 %
5.24 %
1.61 %
1.43 %
Before
After
1.02 %
0.13 %
0.46 %
0.04 %
CD4+ OT-2 T cells
Supplementary Figure S1. Expression of activation markers on T cells before and after isolation of naïve T cells.
Naïve T cells were enriched by negative selections against CD11b, CD19, CD25, CD44, CD49b, F4/80, B220, MHCII, and CD4 or CD8 from the splenocytes of OT-1 or OT-2 mice. Purities for surface markers before and after isolation of naïve OT-1 (upper panels) and OT-2 (lower panels) T cells are shown.

## Slide 2
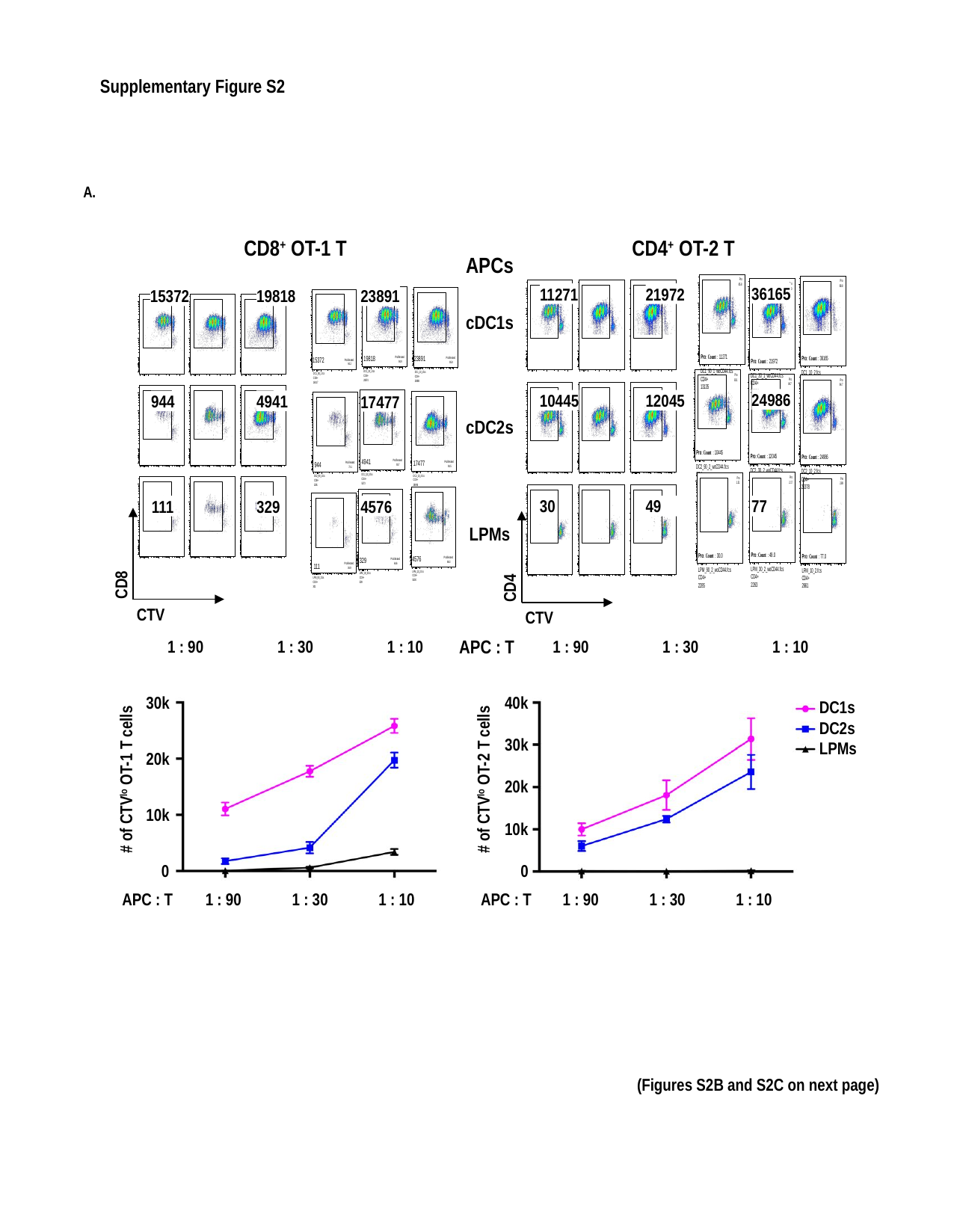

Supplementary Figure S2
A.
CD8+ OT-1 T
CD4+ OT-2 T
APCs
36165
11271
21972
15372
19818
23891
cDC1s
24986
10445
12045
4941
944
17477
cDC2s
30
49
77
111
329
4576
CD8
CTV
CD4
CTV
LPMs
APC : T
1 : 90
1 : 30
1 : 10
1 : 90
1 : 30
1 : 10
30k
40k
30k
# of CTVlo OT-2 T cells
20k
10k
0
1 : 90
1 : 30
1 : 10
DC1s
DC2s
LPMs
20k
# of CTVlo OT-1 T cells
10k
0
APC : T
1 : 90
1 : 30
1 : 10
APC : T
(Figures S2B and S2C on next page)

## Slide 3
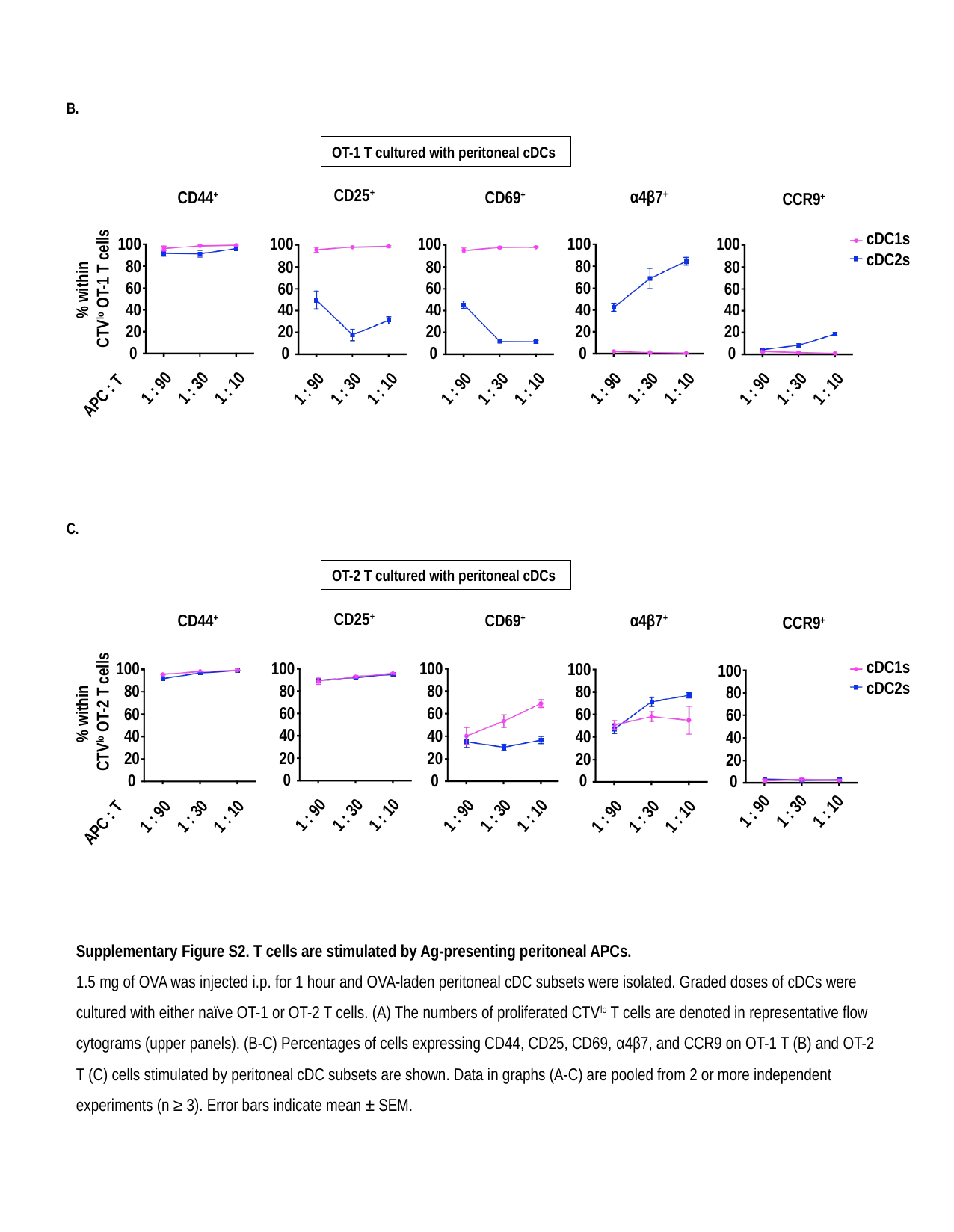

B.
OT-1 T cultured with peritoneal cDCs
CD25+
α4β7+
CD44+
CD69+
CCR9+
cDC1s
cDC2s
100
80
60
40
20
0
1 : 90
1 : 30
1 : 10
100
80
60
40
20
0
1 : 90
1 : 30
1 : 10
100
80
60
40
20
0
1 : 90
1 : 30
1 : 10
100
80
60
40
20
0
1 : 90
1 : 30
1 : 10
100
80
60
40
20
0
1 : 90
1 : 30
1 : 10
% within
 CTVlo OT-1 T cells
APC : T
C.
OT-2 T cultured with peritoneal cDCs
CD25+
α4β7+
CD44+
CD69+
CCR9+
cDC1s
cDC2s
100
80
60
40
20
0
1 : 90
1 : 30
1 : 10
100
80
60
40
20
0
1 : 90
1 : 30
1 : 10
100
80
60
40
20
0
1 : 90
1 : 30
1 : 10
100
80
60
40
20
0
1 : 90
1 : 10
1 : 30
100
80
60
40
20
0
1 : 90
1 : 30
1 : 10
% within
 CTVlo OT-2 T cells
APC : T
Supplementary Figure S2. T cells are stimulated by Ag-presenting peritoneal APCs.
1.5 mg of OVA was injected i.p. for 1 hour and OVA-laden peritoneal cDC subsets were isolated. Graded doses of cDCs were cultured with either naïve OT-1 or OT-2 T cells. (A) The numbers of proliferated CTVlo T cells are denoted in representative flow cytograms (upper panels). (B-C) Percentages of cells expressing CD44, CD25, CD69, α4β7, and CCR9 on OT-1 T (B) and OT-2 T (C) cells stimulated by peritoneal cDC subsets are shown. Data in graphs (A-C) are pooled from 2 or more independent experiments (n ≥ 3). Error bars indicate mean ± SEM.

## Slide 4
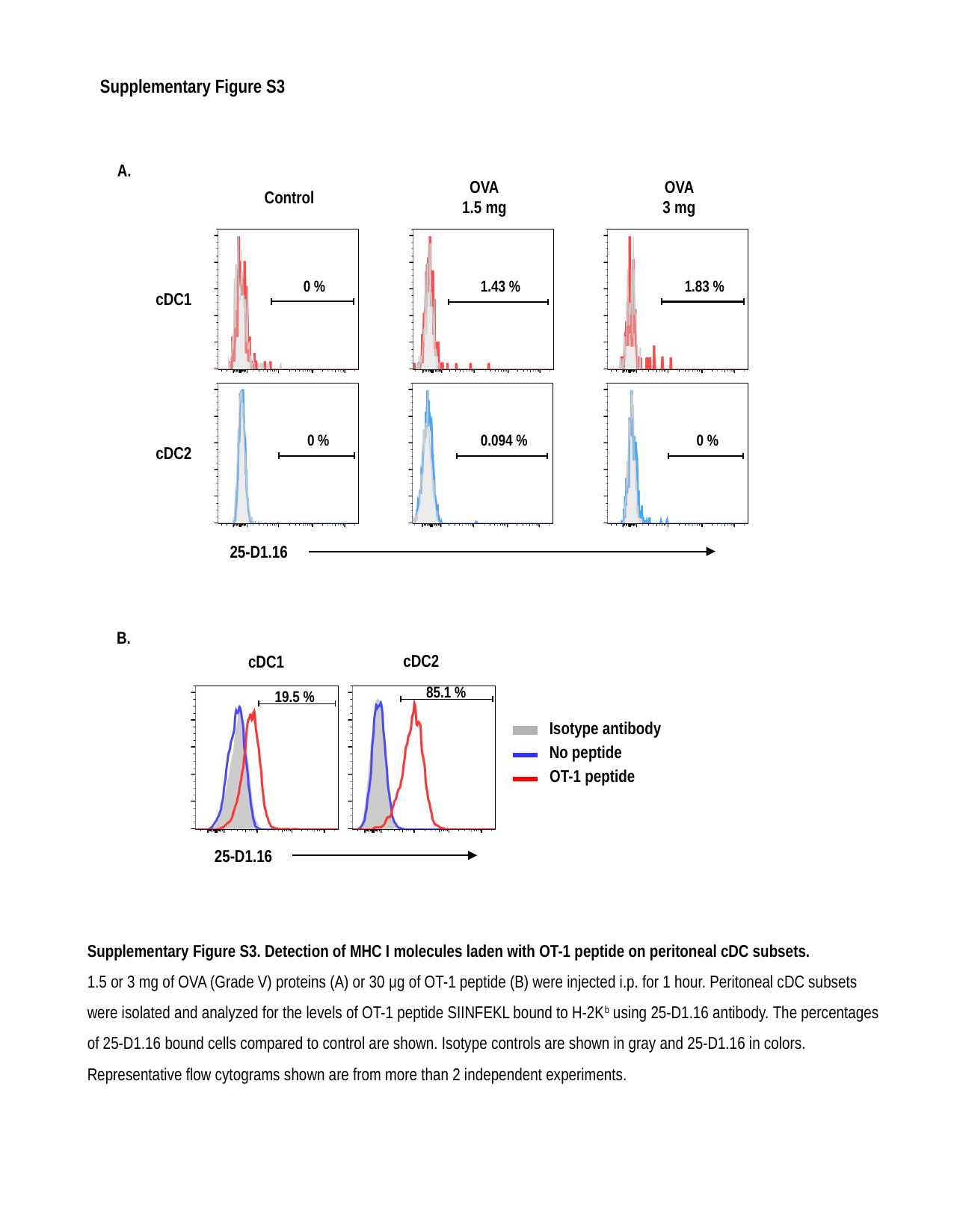

Supplementary Figure S3
A.
OVA
1.5 mg
OVA
3 mg
Control
0 %
1.43 %
1.83 %
cDC1
0 %
0.094 %
0 %
cDC2
25-D1.16
B.
cDC2
cDC1
85.1 %
19.5 %
Isotype antibody
No peptide
OT-1 peptide
25-D1.16
Supplementary Figure S3. Detection of MHC I molecules laden with OT-1 peptide on peritoneal cDC subsets.
1.5 or 3 mg of OVA (Grade V) proteins (A) or 30 μg of OT-1 peptide (B) were injected i.p. for 1 hour. Peritoneal cDC subsets were isolated and analyzed for the levels of OT-1 peptide SIINFEKL bound to H-2Kb using 25-D1.16 antibody. The percentages of 25-D1.16 bound cells compared to control are shown. Isotype controls are shown in gray and 25-D1.16 in colors. Representative flow cytograms shown are from more than 2 independent experiments.

## Slide 5
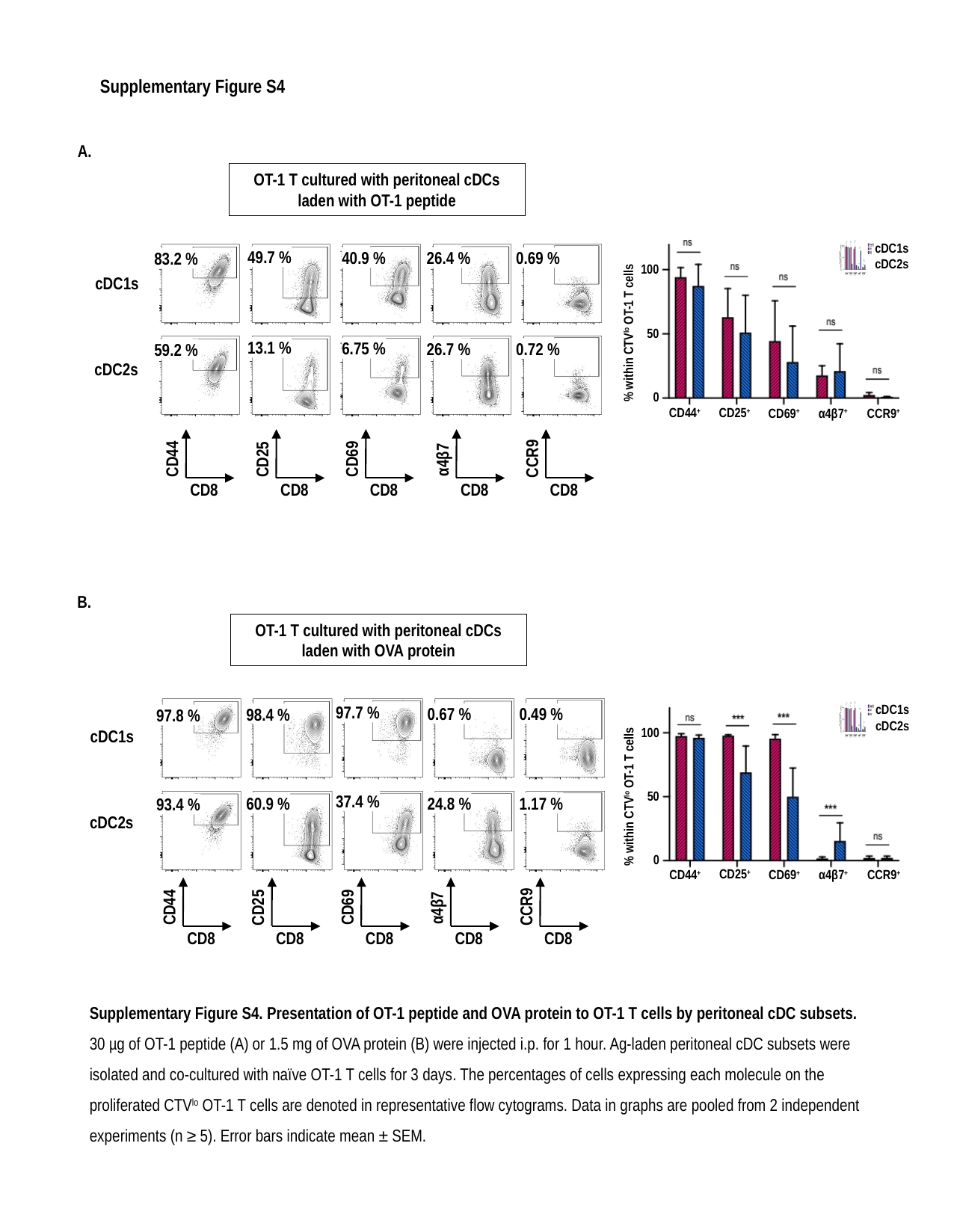

Supplementary Figure S4
A.
OT-1 T cultured with peritoneal cDCs laden with OT-1 peptide
49.7 %
40.9 %
26.4 %
0.69 %
83.2 %
cDC1s
13.1 %
6.75 %
26.7 %
0.72 %
59.2 %
cDC2s
CCR9
CD8
CD44
CD8
CD25
CD8
CD69
CD8
α4β7
CD8
cDC1s
cDC2s
100
% within CTVlo OT-1 T cells
50
0
CD25+
CD44+
CD69+
α4β7+
CCR9+
B.
OT-1 T cultured with peritoneal cDCs laden with OVA protein
97.7 %
0.67 %
0.49 %
98.4 %
97.8 %
cDC1s
37.4 %
24.8 %
1.17 %
60.9 %
93.4 %
cDC2s
CCR9
CD8
CD44
CD8
CD69
CD8
α4β7
CD8
CD25
CD8
cDC1s
cDC2s
100
50
% within CTVlo OT-1 T cells
0
CD25+
CD44+
CD69+
α4β7+
CCR9+
Supplementary Figure S4. Presentation of OT-1 peptide and OVA protein to OT-1 T cells by peritoneal cDC subsets.
30 µg of OT-1 peptide (A) or 1.5 mg of OVA protein (B) were injected i.p. for 1 hour. Ag-laden peritoneal cDC subsets were isolated and co-cultured with naïve OT-1 T cells for 3 days. The percentages of cells expressing each molecule on the proliferated CTVlo OT-1 T cells are denoted in representative flow cytograms. Data in graphs are pooled from 2 independent experiments (n ≥ 5). Error bars indicate mean ± SEM.

## Slide 6
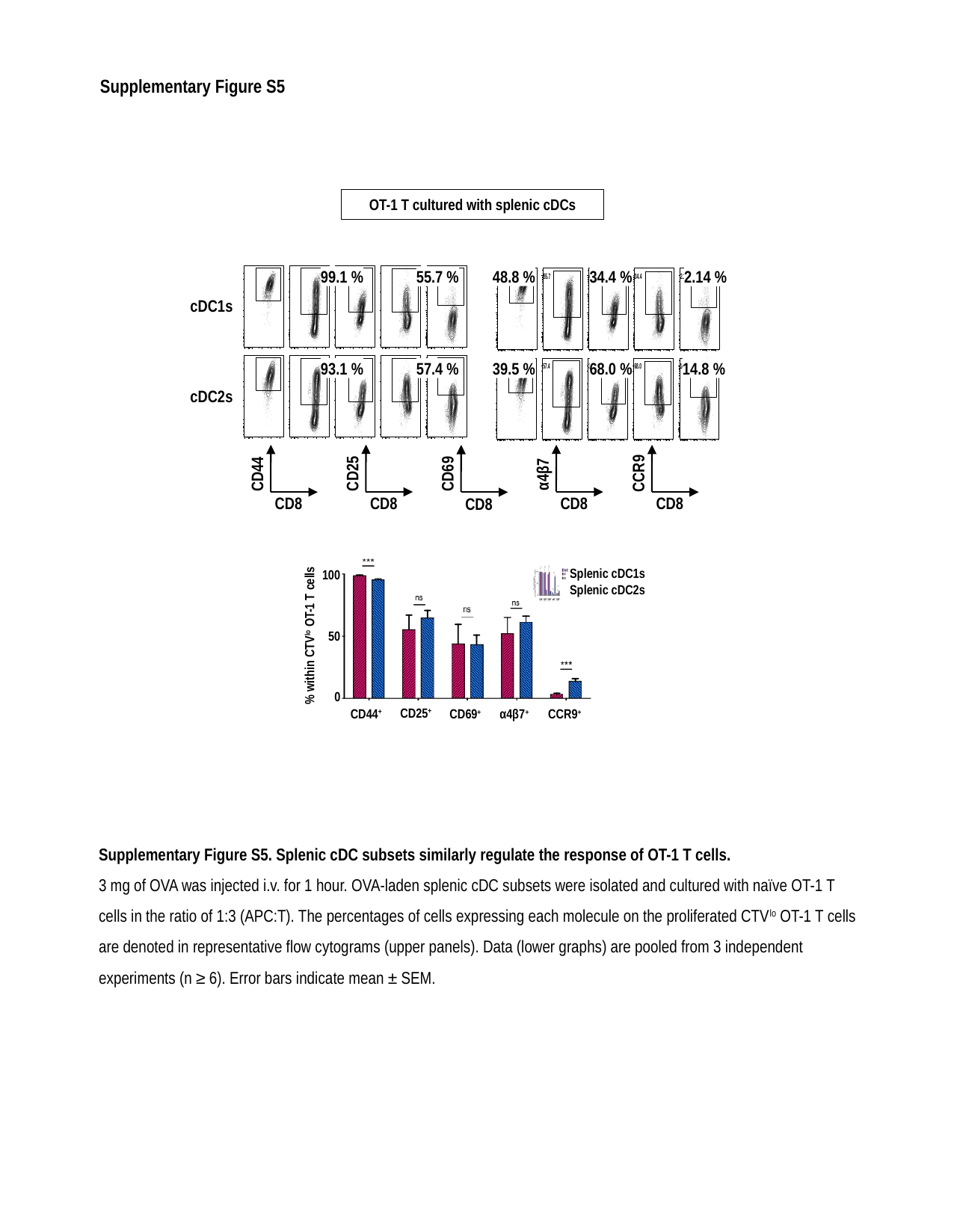

Supplementary Figure S5
OT-1 T cultured with splenic cDCs
48.8 %
99.1 %
34.4 %
55.7 %
2.14 %
cDC1s
93.1 %
57.4 %
39.5 %
68.0 %
14.8 %
cDC2s
CCR9
CD8
CD25
CD8
α4β7
CD8
CD44
CD8
CD69
CD8
Splenic cDC1s
Splenic cDC2s
100
% within CTVlo OT-1 T cells
50
0
CD25+
CD44+
CD69+
α4β7+
CCR9+
Supplementary Figure S5. Splenic cDC subsets similarly regulate the response of OT-1 T cells.
3 mg of OVA was injected i.v. for 1 hour. OVA-laden splenic cDC subsets were isolated and cultured with naïve OT-1 T cells in the ratio of 1:3 (APC:T). The percentages of cells expressing each molecule on the proliferated CTVlo OT-1 T cells are denoted in representative flow cytograms (upper panels). Data (lower graphs) are pooled from 3 independent experiments (n ≥ 6). Error bars indicate mean ± SEM.

## Slide 7
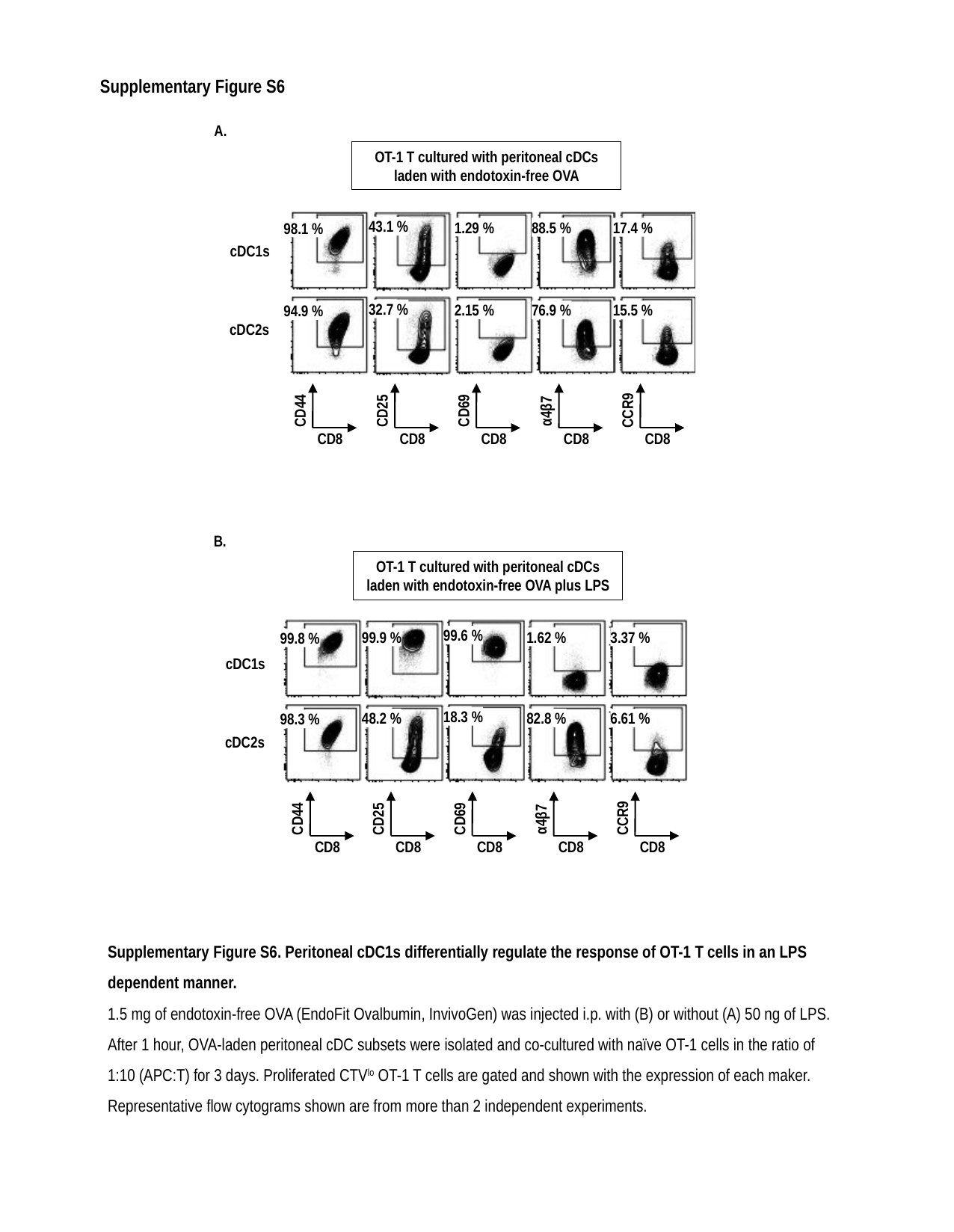

Supplementary Figure S6
A.
OT-1 T cultured with peritoneal cDCs laden with endotoxin-free OVA
43.1 %
1.29 %
88.5 %
17.4 %
98.1 %
cDC1s
32.7 %
2.15 %
76.9 %
15.5 %
94.9 %
cDC2s
CCR9
CD8
CD44
CD8
CD69
CD8
CD25
CD8
α4β7
CD8
B.
OT-1 T cultured with peritoneal cDCs laden with endotoxin-free OVA plus LPS
99.6 %
1.62 %
3.37 %
99.9 %
99.8 %
cDC1s
18.3 %
82.8 %
6.61 %
48.2 %
98.3 %
cDC2s
CCR9
CD8
CD44
CD8
CD69
CD8
CD25
CD8
α4β7
CD8
Supplementary Figure S6. Peritoneal cDC1s differentially regulate the response of OT-1 T cells in an LPS dependent manner.
1.5 mg of endotoxin-free OVA (EndoFit Ovalbumin, InvivoGen) was injected i.p. with (B) or without (A) 50 ng of LPS. After 1 hour, OVA-laden peritoneal cDC subsets were isolated and co-cultured with naïve OT-1 cells in the ratio of 1:10 (APC:T) for 3 days. Proliferated CTVlo OT-1 T cells are gated and shown with the expression of each maker. Representative flow cytograms shown are from more than 2 independent experiments.

## Slide 8
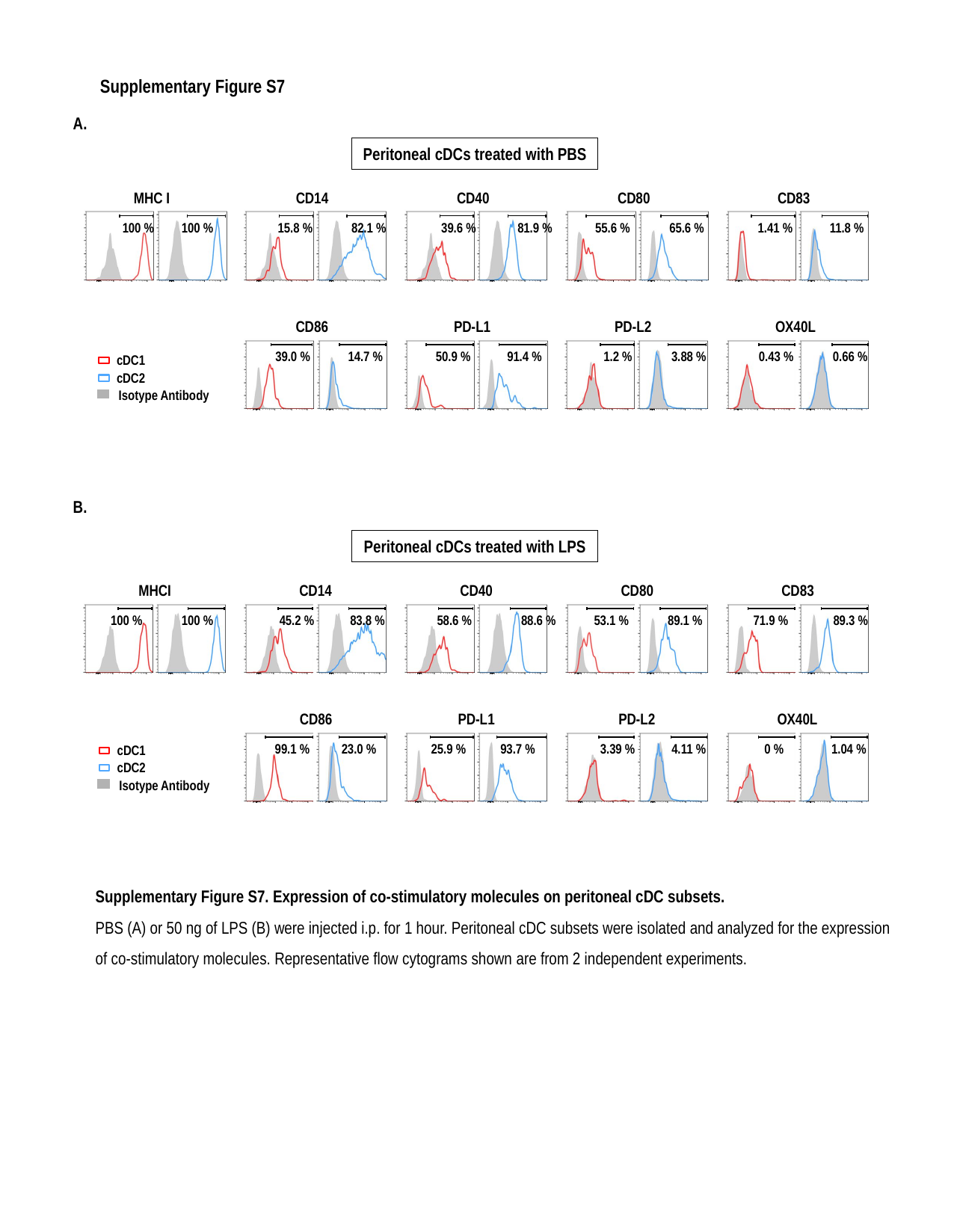

Supplementary Figure S7
A.
Peritoneal cDCs treated with PBS
MHC I
CD14
CD40
CD80
CD83
100 %
100 %
15.8 %
82.1 %
39.6 %
81.9 %
55.6 %
65.6 %
1.41 %
11.8 %
CD86
PD-L1
PD-L2
OX40L
39.0 %
14.7 %
50.9 %
91.4 %
1.2 %
3.88 %
0.43 %
0.66 %
cDC1
cDC2
Isotype Antibody
B.
Peritoneal cDCs treated with LPS
MHCI
CD14
CD40
CD80
CD83
100 %
100 %
45.2 %
83.8 %
58.6 %
88.6 %
53.1 %
89.1 %
71.9 %
89.3 %
CD86
PD-L1
PD-L2
OX40L
99.1 %
23.0 %
25.9 %
93.7 %
3.39 %
4.11 %
0 %
1.04 %
cDC1
cDC2
Isotype Antibody
Supplementary Figure S7. Expression of co-stimulatory molecules on peritoneal cDC subsets.
PBS (A) or 50 ng of LPS (B) were injected i.p. for 1 hour. Peritoneal cDC subsets were isolated and analyzed for the expression of co-stimulatory molecules. Representative flow cytograms shown are from 2 independent experiments.

## Slide 9
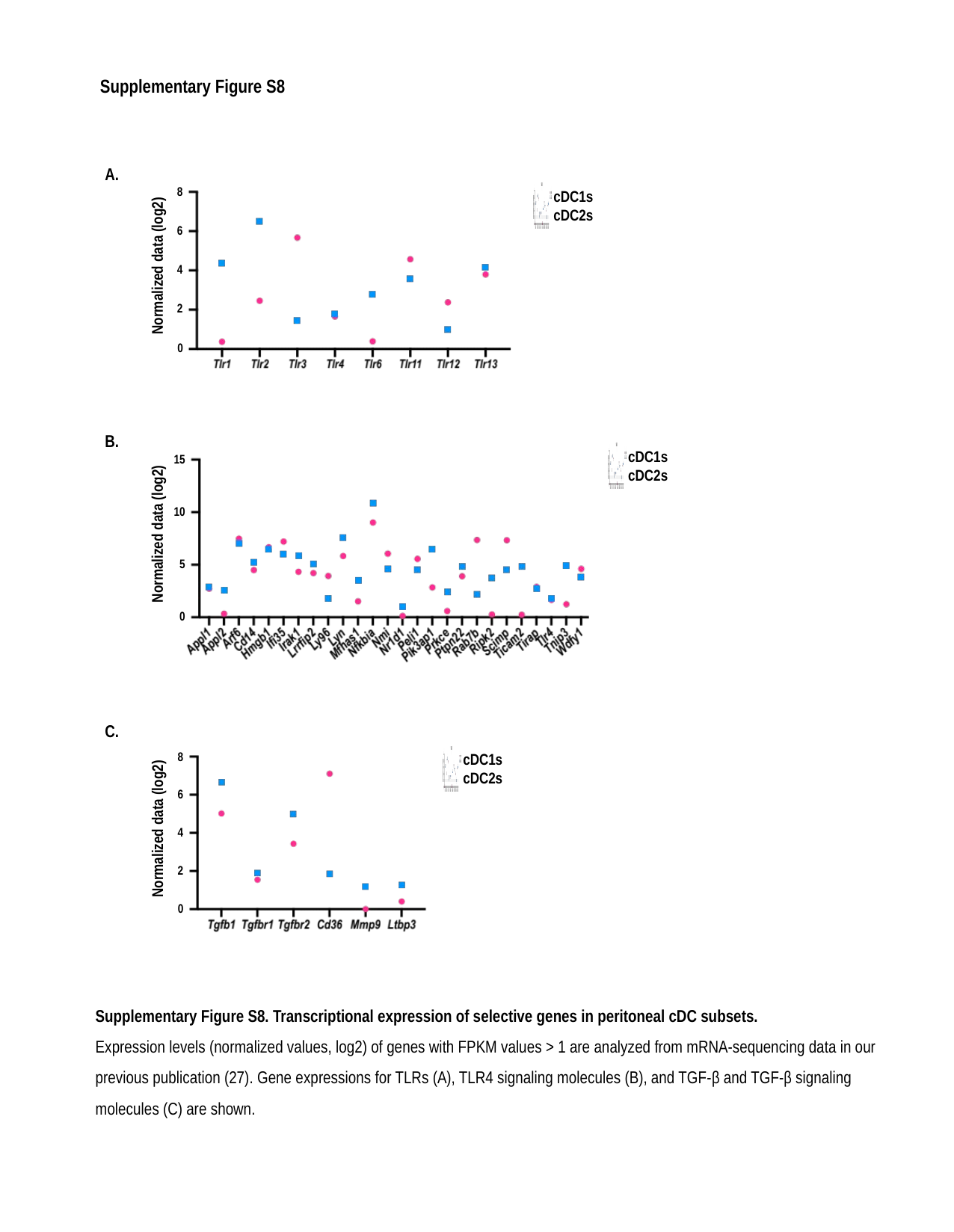

Supplementary Figure S8
A.
8
cDC1s
cDC2s
6
Normalized data (log2)
4
2
0
B.
cDC1s
cDC2s
15
10
Normalized data (log2)
5
0
C.
8
cDC1s
cDC2s
6
Normalized data (log2)
4
2
0
Supplementary Figure S8. Transcriptional expression of selective genes in peritoneal cDC subsets.
Expression levels (normalized values, log2) of genes with FPKM values > 1 are analyzed from mRNA-sequencing data in our previous publication (27). Gene expressions for TLRs (A), TLR4 signaling molecules (B), and TGF-β and TGF-β signaling molecules (C) are shown.

## Slide 10
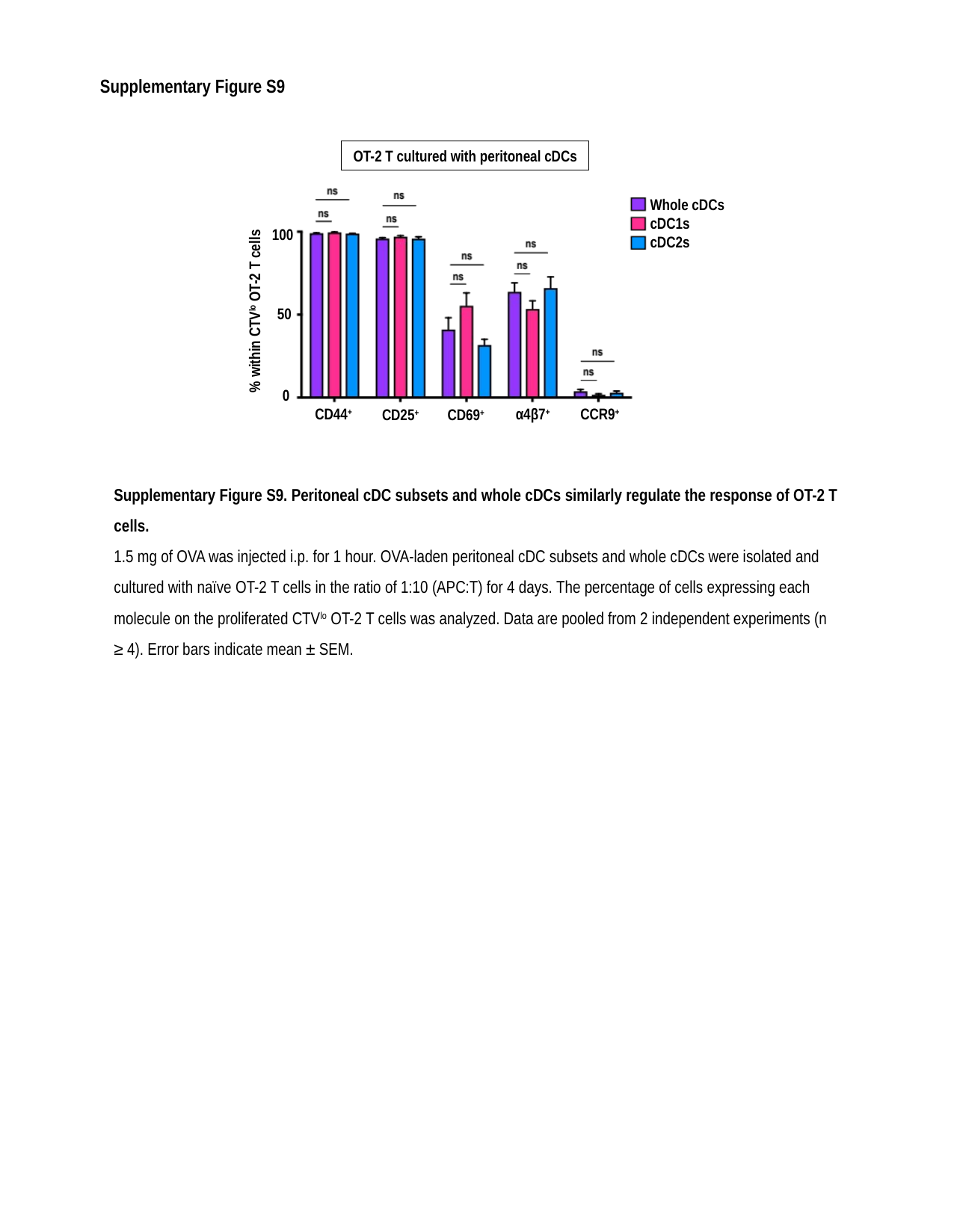

Supplementary Figure S9
OT-2 T cultured with peritoneal cDCs
Whole cDCs
cDC1s
cDC2s
100
% within CTVlo OT-2 T cells
50
0
CD44+
α4β7+
CCR9+
CD25+
CD69+
Supplementary Figure S9. Peritoneal cDC subsets and whole cDCs similarly regulate the response of OT-2 T cells.
1.5 mg of OVA was injected i.p. for 1 hour. OVA-laden peritoneal cDC subsets and whole cDCs were isolated and cultured with naïve OT-2 T cells in the ratio of 1:10 (APC:T) for 4 days. The percentage of cells expressing each molecule on the proliferated CTVlo OT-2 T cells was analyzed. Data are pooled from 2 independent experiments (n ≥ 4). Error bars indicate mean ± SEM.

## Slide 11
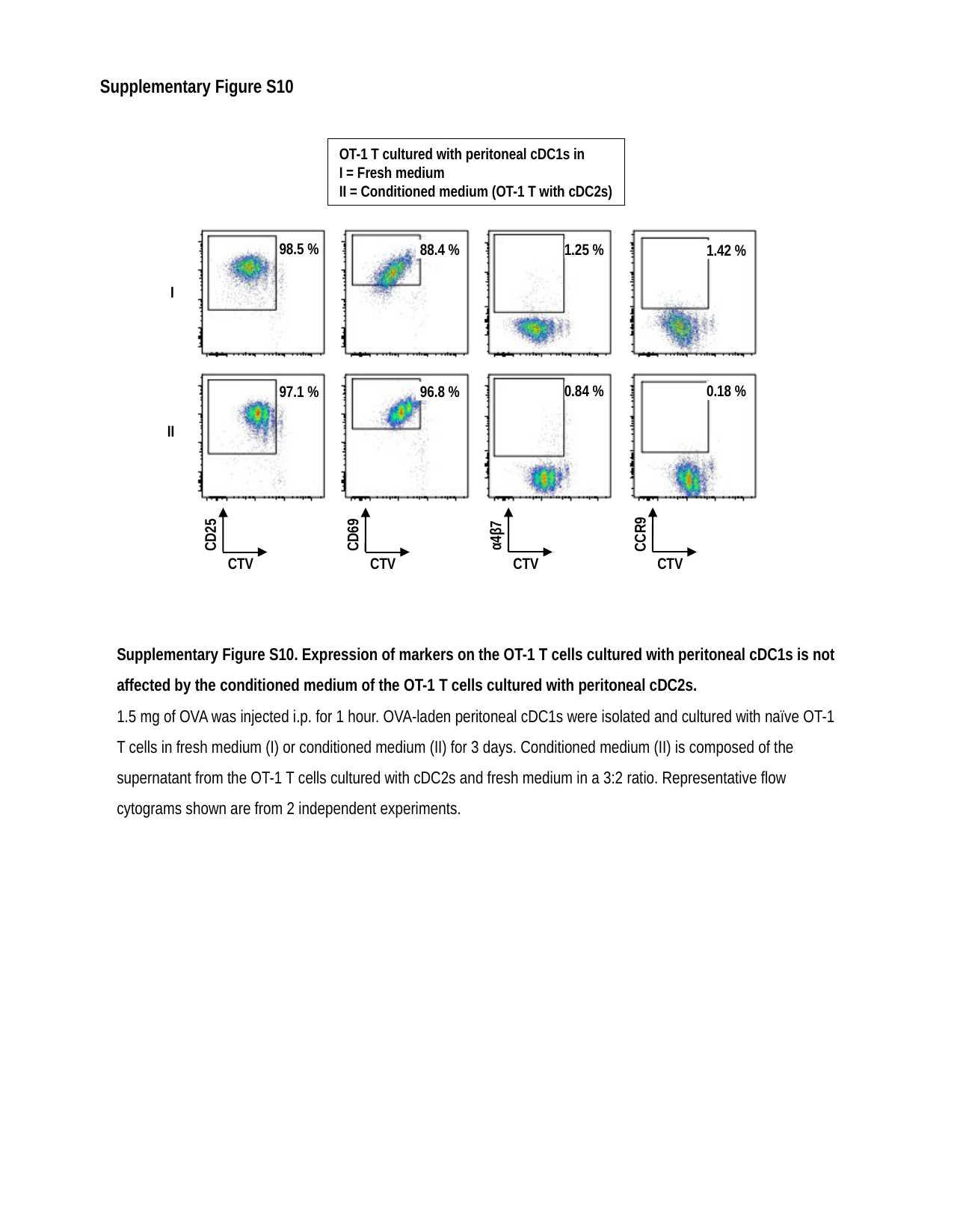

Supplementary Figure S10
OT-1 T cultured with peritoneal cDC1s in
I = Fresh medium
II = Conditioned medium (OT-1 T with cDC2s)
98.5 %
1.25 %
88.4 %
1.42 %
I
0.84 %
0.18 %
97.1 %
96.8 %
II
CCR9
CTV
CD69
CTV
CD25
CTV
α4β7
CTV
Supplementary Figure S10. Expression of markers on the OT-1 T cells cultured with peritoneal cDC1s is not affected by the conditioned medium of the OT-1 T cells cultured with peritoneal cDC2s.
1.5 mg of OVA was injected i.p. for 1 hour. OVA-laden peritoneal cDC1s were isolated and cultured with naïve OT-1 T cells in fresh medium (I) or conditioned medium (II) for 3 days. Conditioned medium (II) is composed of the supernatant from the OT-1 T cells cultured with cDC2s and fresh medium in a 3:2 ratio. Representative flow cytograms shown are from 2 independent experiments.

## Slide 12
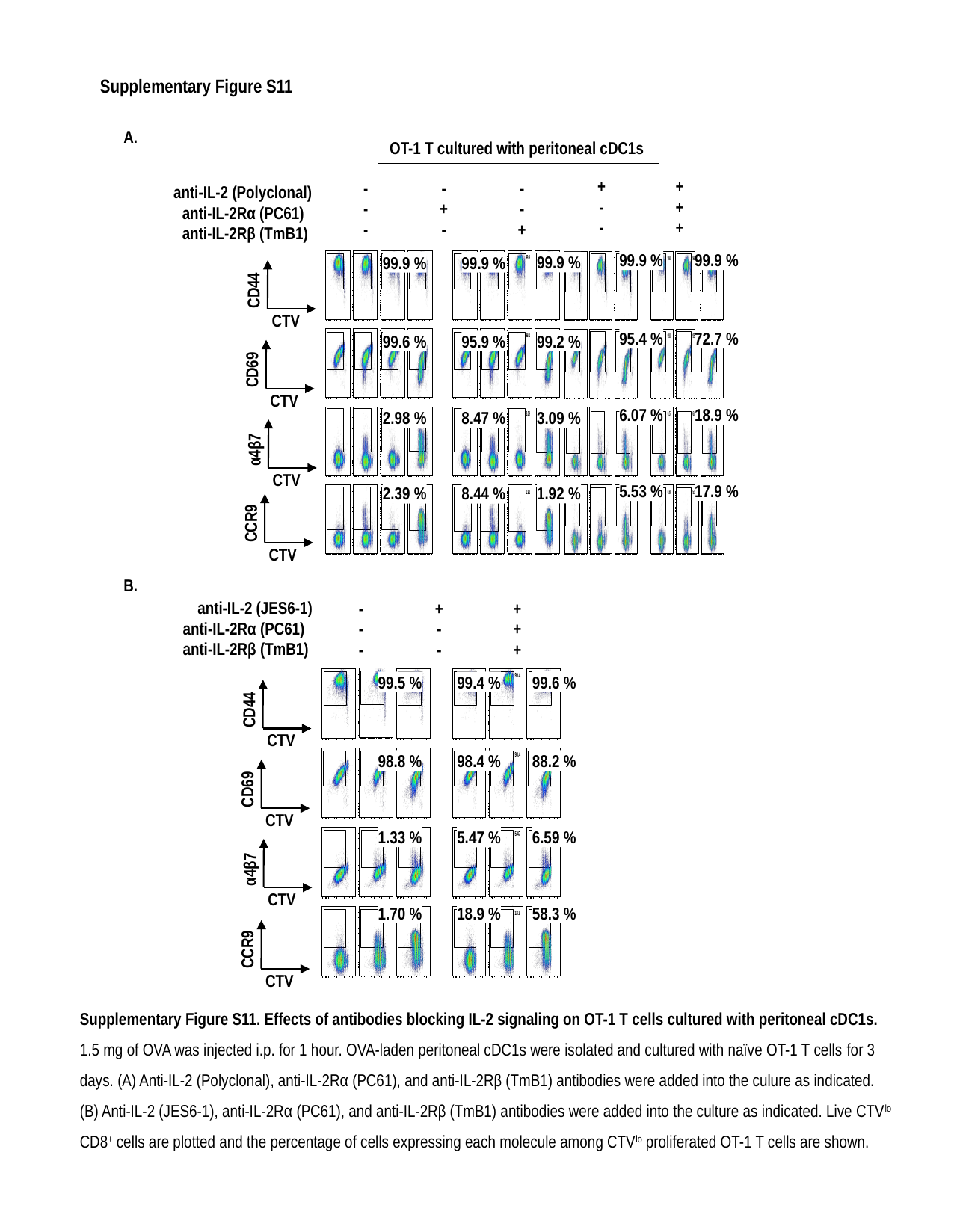

Supplementary Figure S11
A.
OT-1 T cultured with peritoneal cDC1s
+
-
-
+
+
+
-
+
-
-
-
+
-
-
-
anti-IL-2 (Polyclonal)
anti-IL-2Rα (PC61)
anti-IL-2Rβ (TmB1)
99.9 %
99.9 %
99.9 %
99.9 %
99.9 %
CD44
CTV
72.7 %
95.4 %
99.2 %
99.6 %
95.9 %
CD69
CTV
18.9 %
6.07 %
3.09 %
2.98 %
8.47 %
α4β7
CTV
17.9 %
5.53 %
1.92 %
2.39 %
8.44 %
CCR9
CTV
B.
anti-IL-2 (JES6-1)
anti-IL-2Rα (PC61)
anti-IL-2Rβ (TmB1)
+
-
-
+
+
+
-
-
-
99.6 %
99.5 %
99.4 %
CD44
CTV
88.2 %
98.8 %
98.4 %
CD69
CTV
6.59 %
1.33 %
5.47 %
α4β7
CTV
58.3 %
1.70 %
18.9 %
CCR9
CTV
Supplementary Figure S11. Effects of antibodies blocking IL-2 signaling on OT-1 T cells cultured with peritoneal cDC1s.
1.5 mg of OVA was injected i.p. for 1 hour. OVA-laden peritoneal cDC1s were isolated and cultured with naïve OT-1 T cells for 3 days. (A) Anti-IL-2 (Polyclonal), anti-IL-2Rα (PC61), and anti-IL-2Rβ (TmB1) antibodies were added into the culure as indicated. (B) Anti-IL-2 (JES6-1), anti-IL-2Rα (PC61), and anti-IL-2Rβ (TmB1) antibodies were added into the culture as indicated. Live CTVlo CD8+ cells are plotted and the percentage of cells expressing each molecule among CTVlo proliferated OT-1 T cells are shown.

## Slide 13
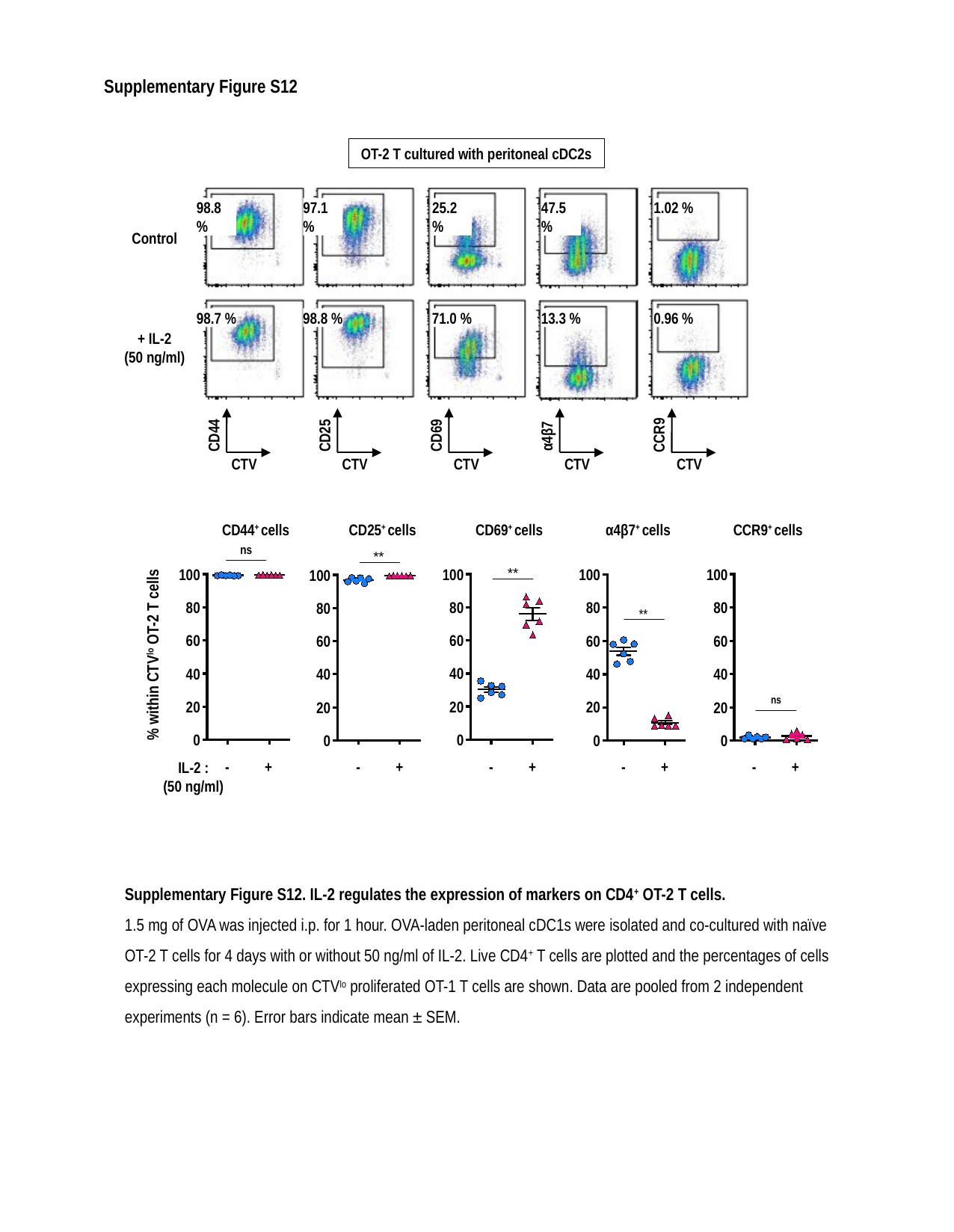

Supplementary Figure S12
OT-2 T cultured with peritoneal cDC2s
98.8 %
97.1 %
25.2 %
47.5 %
1.02 %
Control
98.7 %
98.8 %
71.0 %
13.3 %
0.96 %
+ IL-2
(50 ng/ml)
CCR9
CTV
CD44
CTV
CD69
CTV
CD25
CTV
α4β7
CTV
CD44+ cells
CD25+ cells
CD69+ cells
 α4β7+ cells
CCR9+ cells
ns
100
80
60
40
20
0
-
+
**
100
80
60
40
20
0
-
+
100
**
80
60
40
20
0
-
+
100
80
60
40
20
ns
0
-
+
100
80
**
60
40
20
0
-
+
% within CTVlo OT-2 T cells
IL-2 :
(50 ng/ml)
Supplementary Figure S12. IL-2 regulates the expression of markers on CD4+ OT-2 T cells.
1.5 mg of OVA was injected i.p. for 1 hour. OVA-laden peritoneal cDC1s were isolated and co-cultured with naïve OT-2 T cells for 4 days with or without 50 ng/ml of IL-2. Live CD4+ T cells are plotted and the percentages of cells expressing each molecule on CTVlo proliferated OT-1 T cells are shown. Data are pooled from 2 independent experiments (n = 6). Error bars indicate mean ± SEM.

## Slide 14
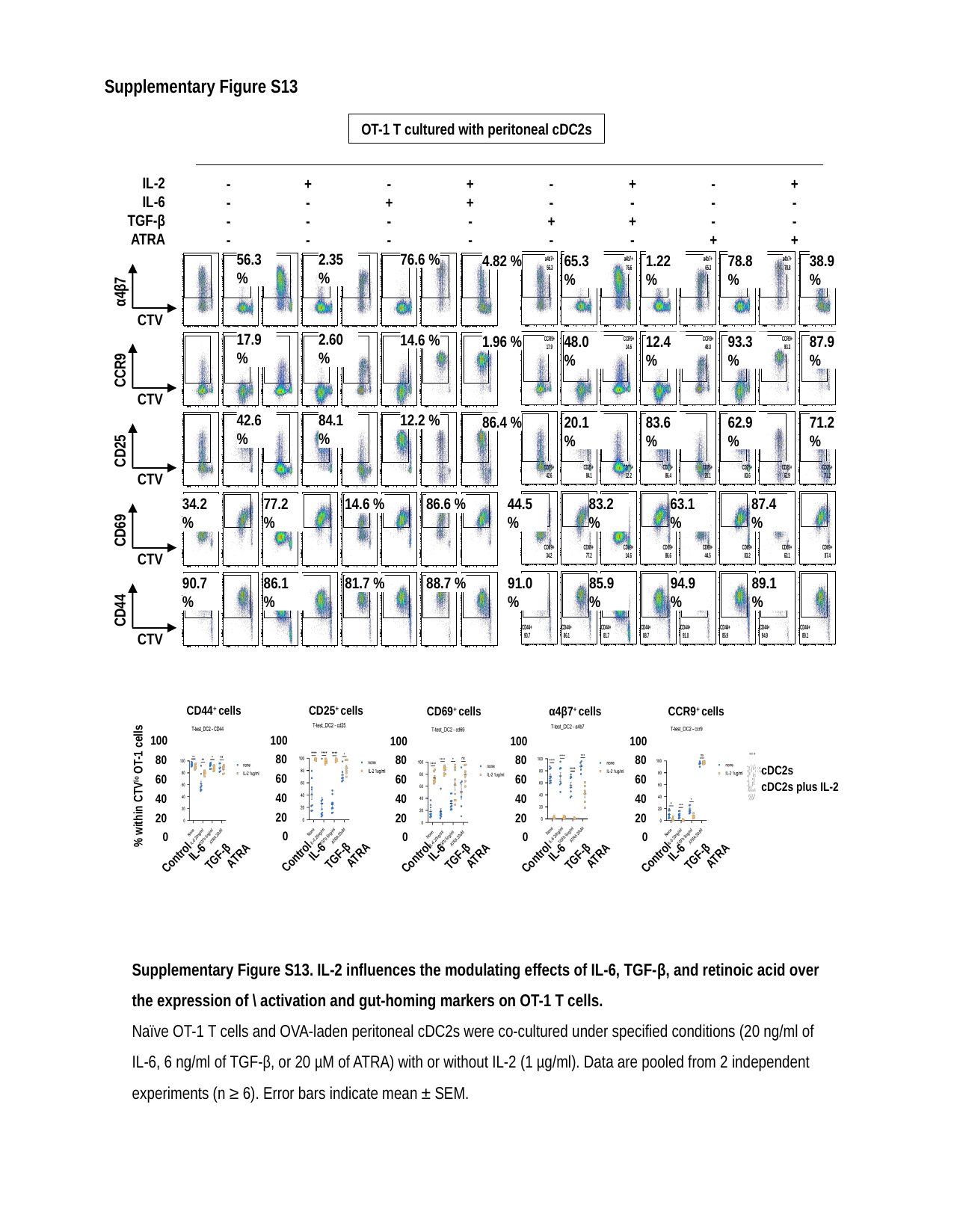

Supplementary Figure S13
OT-1 T cultured with peritoneal cDC2s
IL-2
IL-6
TGF-β
ATRA
-
-
-
-
+
-
-
-
-
+
-
-
+
+
-
-
-
-
+
-
+
-
+
-
-
-
-
+
+
-
-
+
56.3 %
2.35 %
76.6 %
4.82 %
65.3 %
1.22 %
78.8 %
38.9 %
α4β7
CTV
17.9 %
2.60 %
14.6 %
1.96 %
48.0 %
12.4 %
93.3 %
87.9 %
CCR9
CTV
42.6 %
84.1 %
12.2 %
86.4 %
20.1 %
83.6 %
62.9 %
71.2 %
CD25
CTV
34.2 %
77.2 %
14.6 %
86.6 %
44.5 %
83.2 %
63.1 %
87.4 %
CD69
CTV
90.7 %
86.1 %
81.7 %
88.7 %
91.0 %
85.9 %
94.9 %
89.1 %
CD44
CTV
CD44+ cells
0
IL-6
CD25+ cells
100
80
60
40
20
0
IL-6
ATRA
TGF-β
Control
CD69+ cells
100
80
60
40
20
0
IL-6
ATRA
TGF-β
Control
 α4β7+ cells
100
80
60
40
20
0
IL-6
ATRA
TGF-β
Control
CCR9+ cells
100
80
60
40
20
0
IL-6
ATRA
TGF-β
Control
100
80
cDC2s
cDC2s plus IL-2
60
% within CTVlo OT-1 cells
40
20
ATRA
TGF-β
Control
Supplementary Figure S13. IL-2 influences the modulating effects of IL-6, TGF-β, and retinoic acid over the expression of \ activation and gut-homing markers on OT-1 T cells.
Naïve OT-1 T cells and OVA-laden peritoneal cDC2s were co-cultured under specified conditions (20 ng/ml of IL-6, 6 ng/ml of TGF-β, or 20 µM of ATRA) with or without IL-2 (1 µg/ml). Data are pooled from 2 independent experiments (n ≥ 6). Error bars indicate mean ± SEM.

## Slide 15
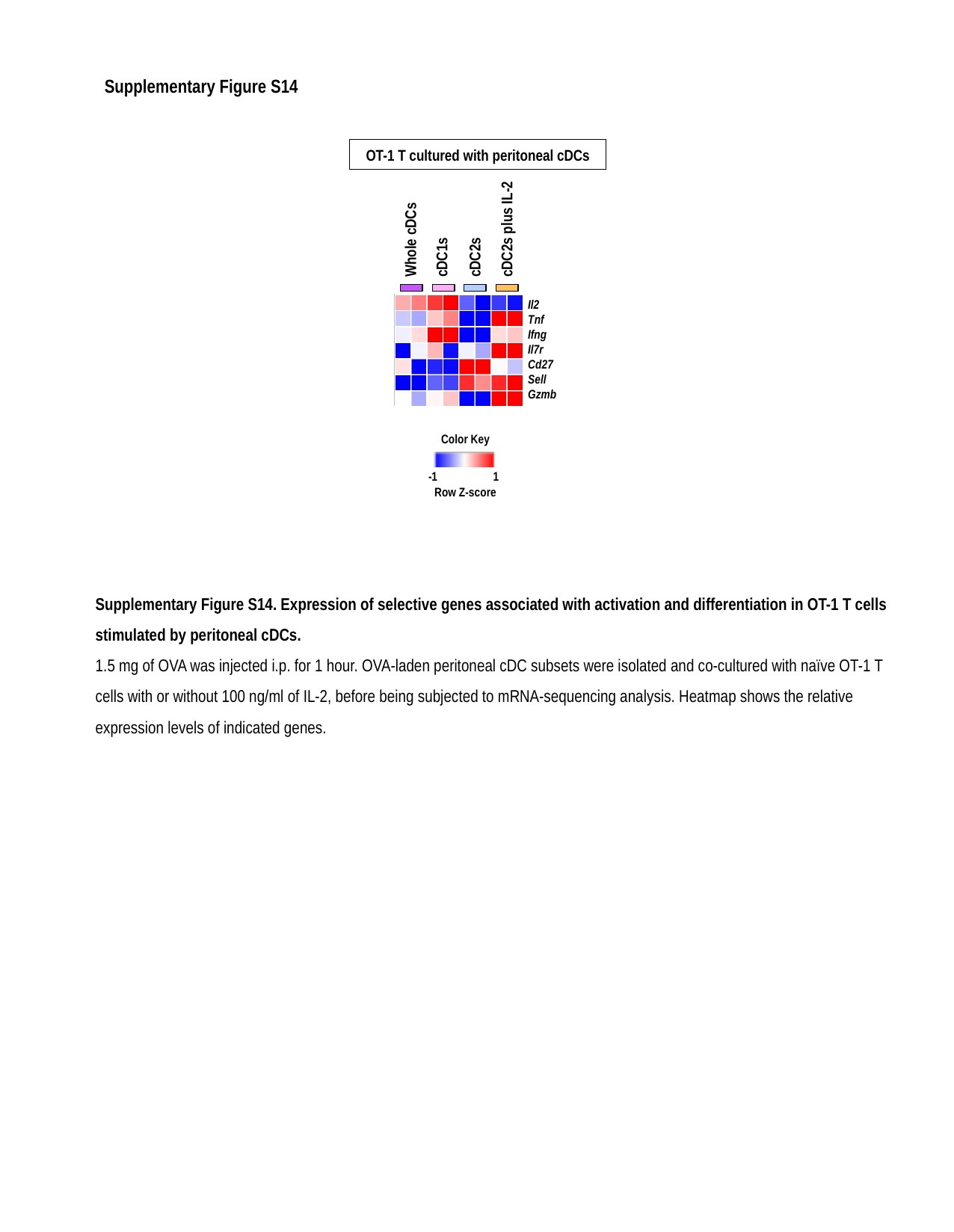

Supplementary Figure S14
OT-1 T cultured with peritoneal cDCs
cDC2s plus IL-2
Whole cDCs
cDC1s
cDC2s
Il2
Tnf
Ifng
Il7r
Cd27
Sell
Gzmb
Color Key
-1
1
Row Z-score
Supplementary Figure S14. Expression of selective genes associated with activation and differentiation in OT-1 T cells stimulated by peritoneal cDCs.
1.5 mg of OVA was injected i.p. for 1 hour. OVA-laden peritoneal cDC subsets were isolated and co-cultured with naïve OT-1 T cells with or without 100 ng/ml of IL-2, before being subjected to mRNA-sequencing analysis. Heatmap shows the relative expression levels of indicated genes.

## Slide 16
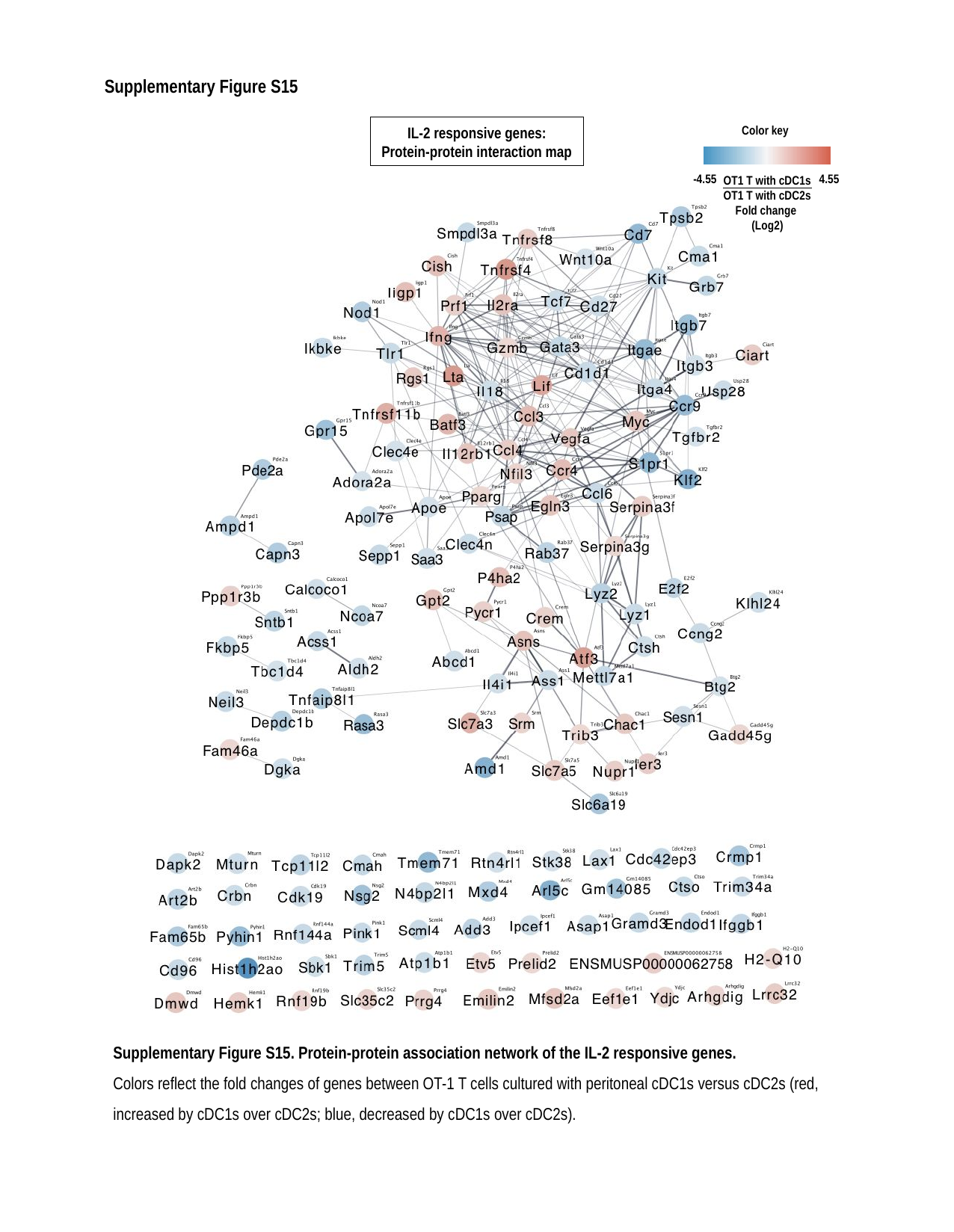

Supplementary Figure S15
Color key
-4.55
4.55
OT1 T with cDC1s
OT1 T with cDC2s
Fold change
(Log2)
IL-2 responsive genes:
Protein-protein interaction map
Supplementary Figure S15. Protein-protein association network of the IL-2 responsive genes.
Colors reflect the fold changes of genes between OT-1 T cells cultured with peritoneal cDC1s versus cDC2s (red, increased by cDC1s over cDC2s; blue, decreased by cDC1s over cDC2s).

## Slide 17
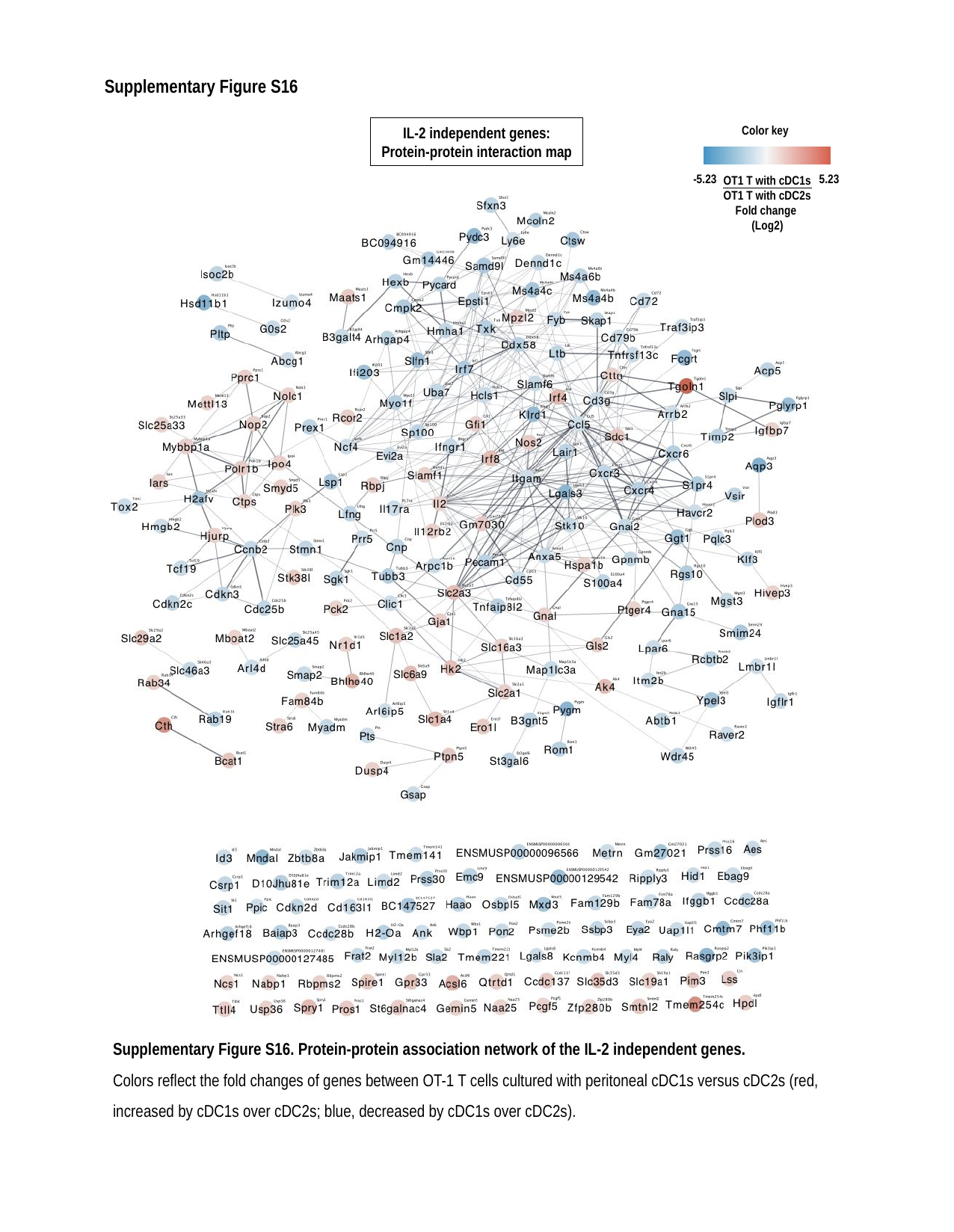

Supplementary Figure S16
Color key
-5.23
5.23
OT1 T with cDC1s
OT1 T with cDC2s
Fold change
(Log2)
IL-2 independent genes:
Protein-protein interaction map
Supplementary Figure S16. Protein-protein association network of the IL-2 independent genes.
Colors reflect the fold changes of genes between OT-1 T cells cultured with peritoneal cDC1s versus cDC2s (red, increased by cDC1s over cDC2s; blue, decreased by cDC1s over cDC2s).

## Slide 18
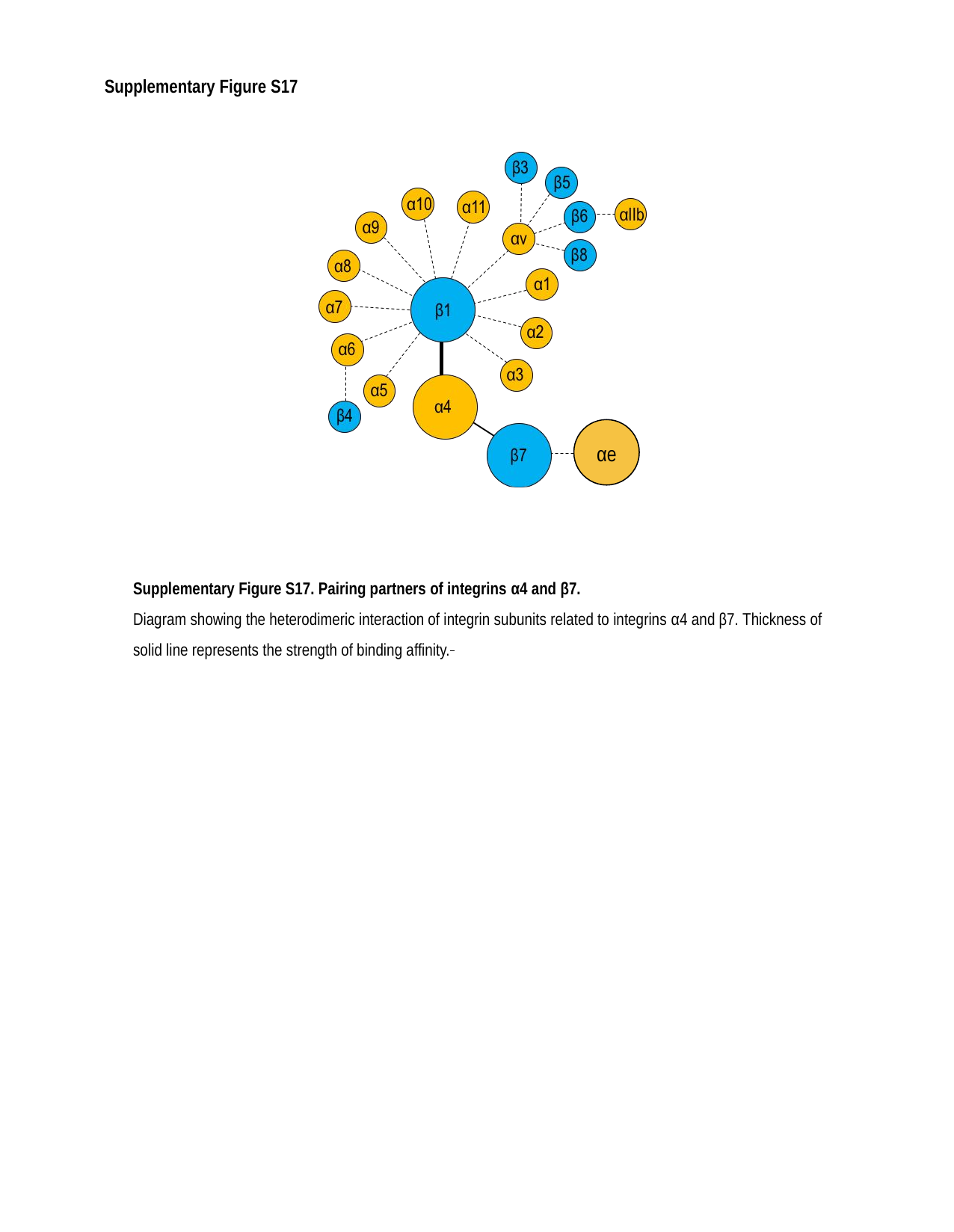

Supplementary Figure S17
αe
Supplementary Figure S17. Pairing partners of integrins α4 and β7.
Diagram showing the heterodimeric interaction of integrin subunits related to integrins α4 and β7. Thickness of solid line represents the strength of binding affinity.

## Slide 19
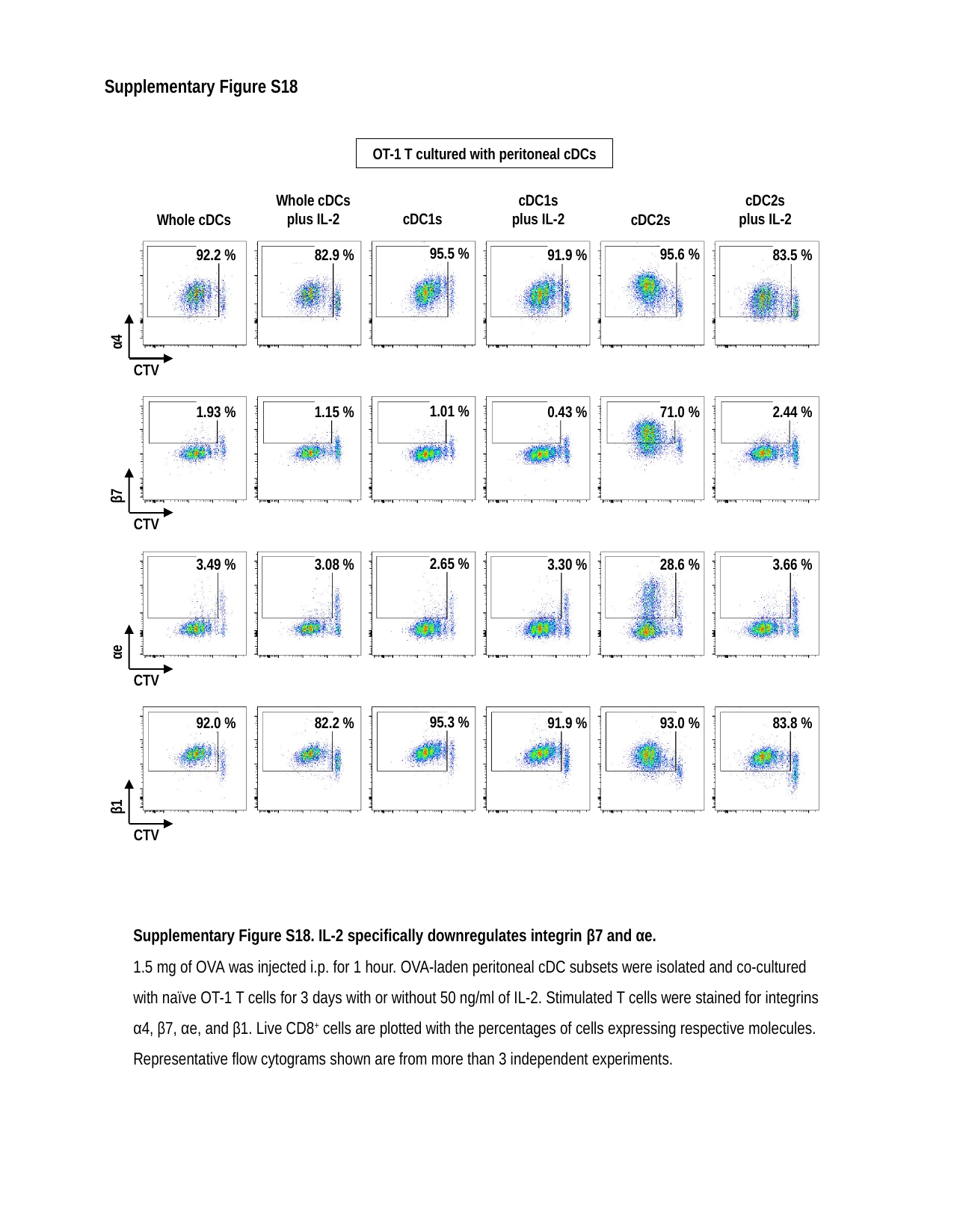

Supplementary Figure S18
OT-1 T cultured with peritoneal cDCs
Whole cDCs
plus IL-2
cDC1s
plus IL-2
cDC2s
plus IL-2
cDC1s
Whole cDCs
cDC2s
95.5 %
95.6 %
92.2 %
82.9 %
91.9 %
83.5 %
α4
CTV
1.01 %
1.93 %
1.15 %
0.43 %
71.0 %
2.44 %
β7
CTV
2.65 %
3.49 %
3.08 %
3.30 %
28.6 %
3.66 %
αe
CTV
95.3 %
92.0 %
82.2 %
91.9 %
93.0 %
83.8 %
β1
CTV
Supplementary Figure S18. IL-2 specifically downregulates integrin β7 and αe.
1.5 mg of OVA was injected i.p. for 1 hour. OVA-laden peritoneal cDC subsets were isolated and co-cultured with naïve OT-1 T cells for 3 days with or without 50 ng/ml of IL-2. Stimulated T cells were stained for integrins α4, β7, αe, and β1. Live CD8+ cells are plotted with the percentages of cells expressing respective molecules. Representative flow cytograms shown are from more than 3 independent experiments.

## Slide 20
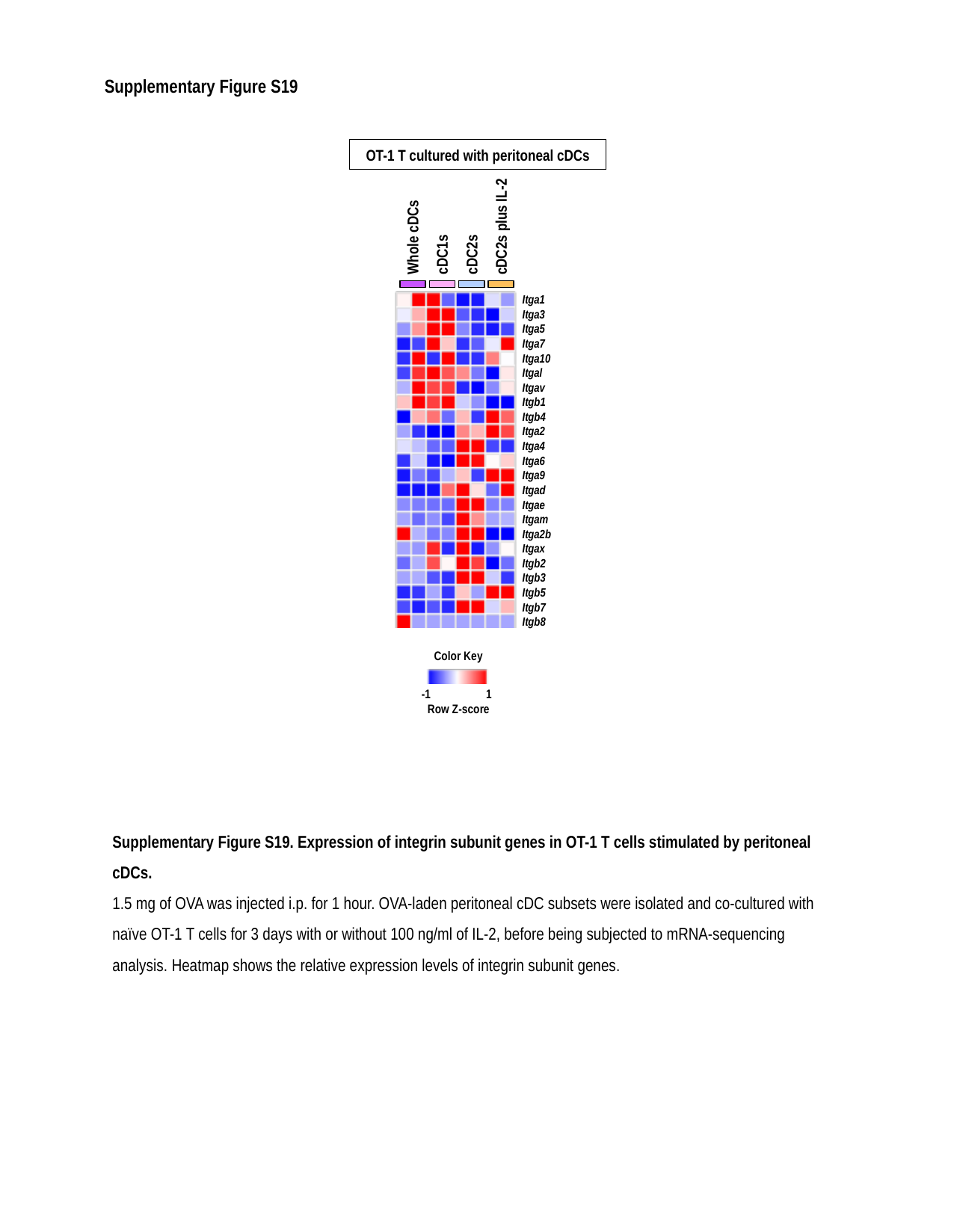

Supplementary Figure S19
OT-1 T cultured with peritoneal cDCs
cDC2s plus IL-2
Whole cDCs
cDC1s
cDC2s
Itga1
Itga3
Itga5
Itga7
Itga10
Itgal
Itgav
Itgb1
Itgb4
Itga2
Itga4
Itga6
Itga9
Itgad
Itgae
Itgam
Itga2b
Itgax
Itgb2
Itgb3
Itgb5
Itgb7
Itgb8
Color Key
-1
1
Row Z-score
Supplementary Figure S19. Expression of integrin subunit genes in OT-1 T cells stimulated by peritoneal cDCs.
1.5 mg of OVA was injected i.p. for 1 hour. OVA-laden peritoneal cDC subsets were isolated and co-cultured with naïve OT-1 T cells for 3 days with or without 100 ng/ml of IL-2, before being subjected to mRNA-sequencing analysis. Heatmap shows the relative expression levels of integrin subunit genes.

## Slide 21
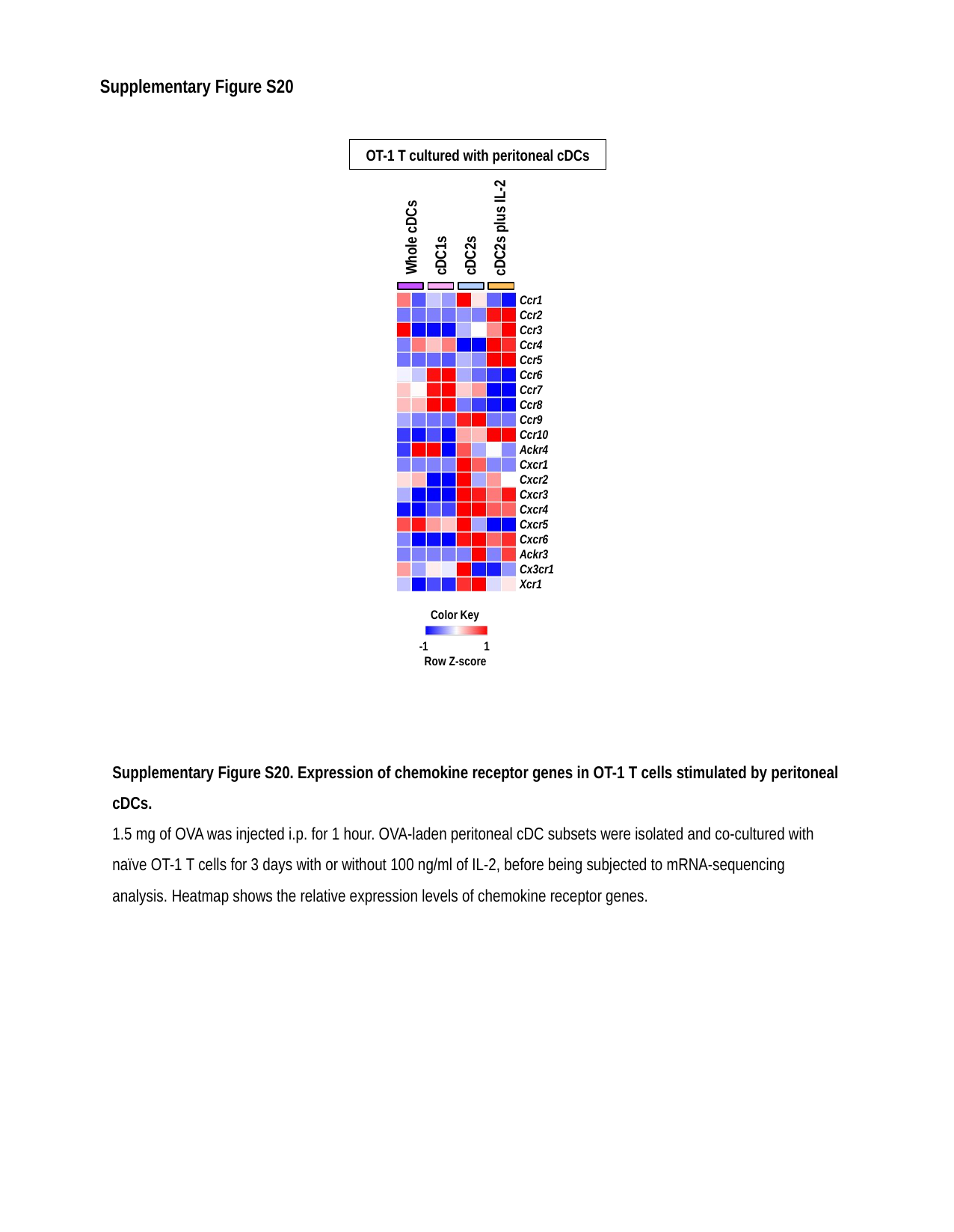

Supplementary Figure S20
OT-1 T cultured with peritoneal cDCs
cDC2s plus IL-2
Whole cDCs
cDC1s
cDC2s
Ccr1
Ccr2
Ccr3
Ccr4
Ccr5
Ccr6
Ccr7
Ccr8
Ccr9
Ccr10
Ackr4
Cxcr1
Cxcr2
Cxcr3
Cxcr4
Cxcr5
Cxcr6
Ackr3
Cx3cr1
Xcr1
Color Key
-1
1
Row Z-score
Supplementary Figure S20. Expression of chemokine receptor genes in OT-1 T cells stimulated by peritoneal cDCs.
1.5 mg of OVA was injected i.p. for 1 hour. OVA-laden peritoneal cDC subsets were isolated and co-cultured with naïve OT-1 T cells for 3 days with or without 100 ng/ml of IL-2, before being subjected to mRNA-sequencing analysis. Heatmap shows the relative expression levels of chemokine receptor genes.

## Slide 22
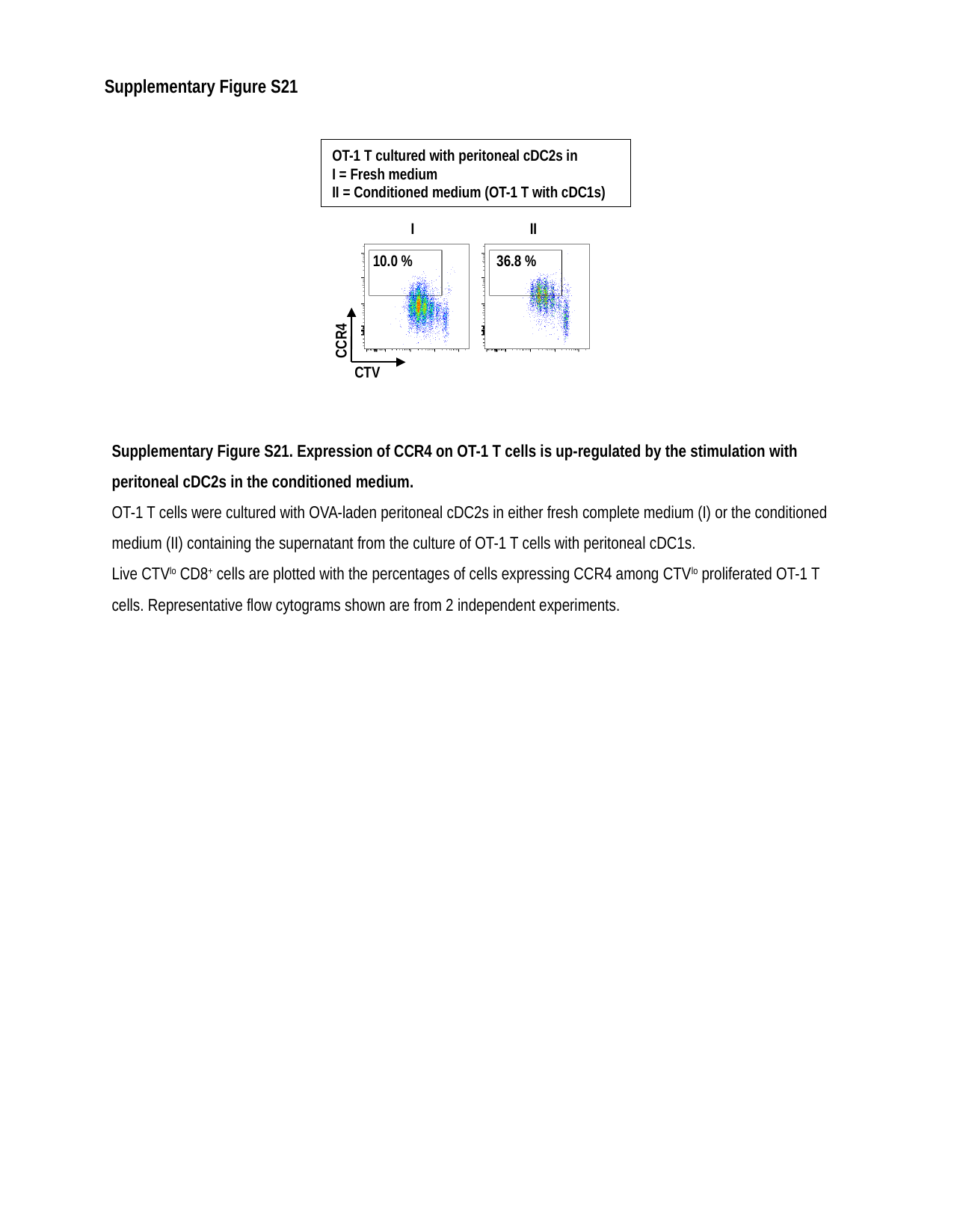

Supplementary Figure S21
OT-1 T cultured with peritoneal cDC2s in
I = Fresh medium
II = Conditioned medium (OT-1 T with cDC1s)
I
II
10.0 %
36.8 %
CCR4
CTV
Supplementary Figure S21. Expression of CCR4 on OT-1 T cells is up-regulated by the stimulation with peritoneal cDC2s in the conditioned medium.
OT-1 T cells were cultured with OVA-laden peritoneal cDC2s in either fresh complete medium (I) or the conditioned medium (II) containing the supernatant from the culture of OT-1 T cells with peritoneal cDC1s.
Live CTVlo CD8+ cells are plotted with the percentages of cells expressing CCR4 among CTVlo proliferated OT-1 T cells. Representative flow cytograms shown are from 2 independent experiments.

## Slide 23
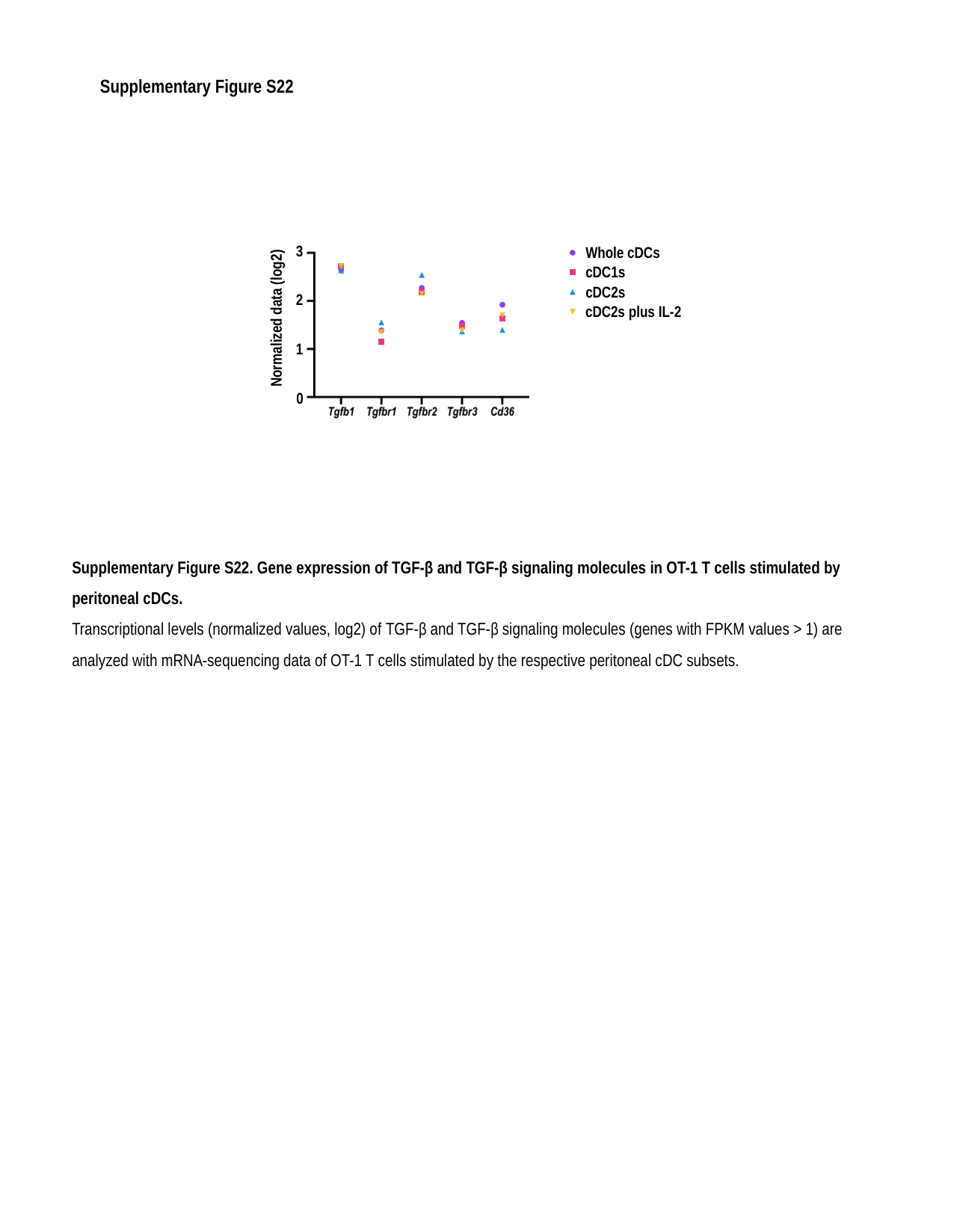

Supplementary Figure S22
3
Whole cDCs
cDC1s
cDC2s
2
cDC2s plus IL-2
Normalized data (log2)
1
0
Supplementary Figure S22. Gene expression of TGF-β and TGF-β signaling molecules in OT-1 T cells stimulated by peritoneal cDCs.
Transcriptional levels (normalized values, log2) of TGF-β and TGF-β signaling molecules (genes with FPKM values > 1) are analyzed with mRNA-sequencing data of OT-1 T cells stimulated by the respective peritoneal cDC subsets.

## Slide 24
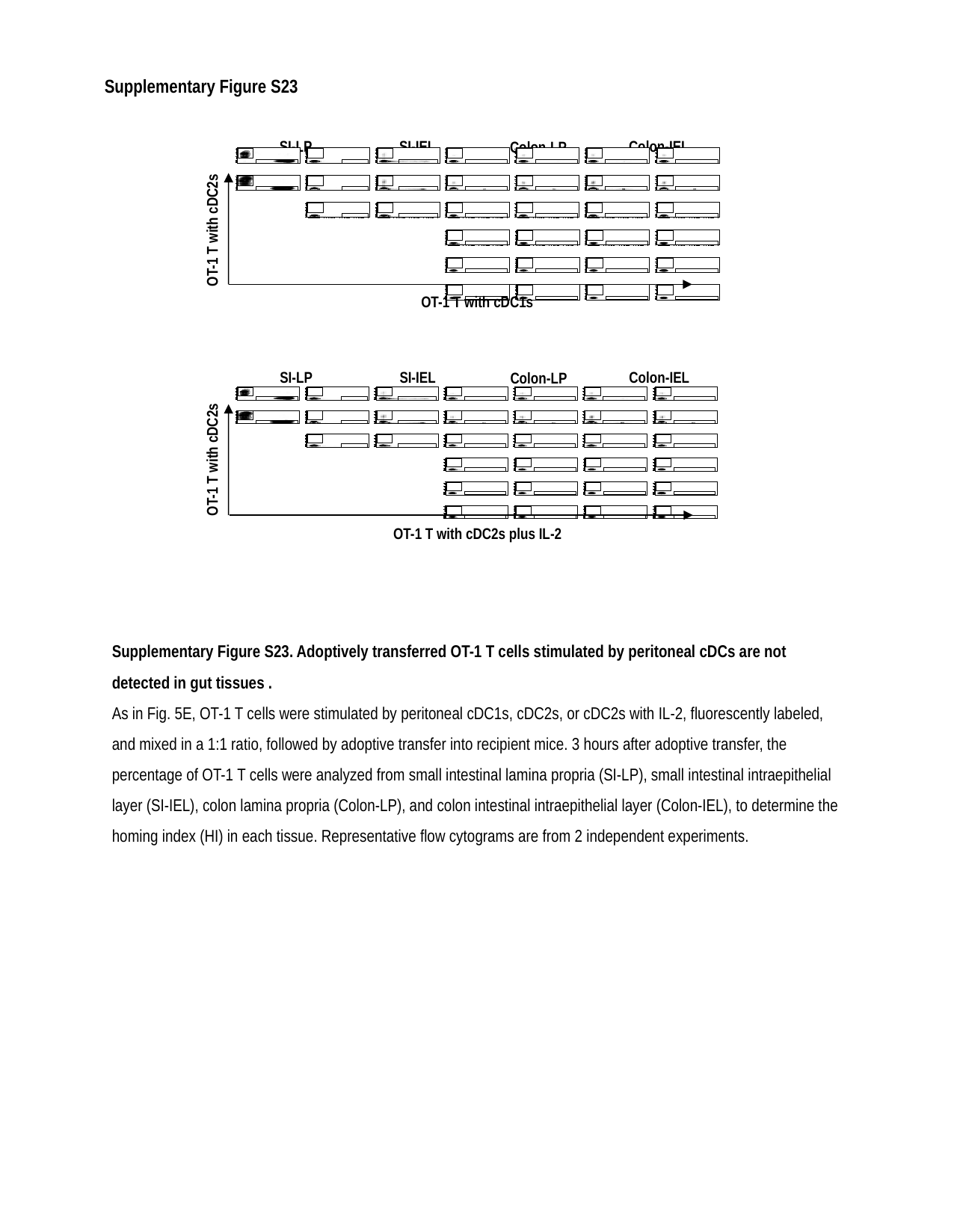

Supplementary Figure S23
SI-IEL
SI-LP
Colon-IEL
Colon-LP
OT-1 T with cDC2s
OT-1 T with cDC1s
SI-IEL
SI-LP
Colon-IEL
Colon-LP
OT-1 T with cDC2s
OT-1 T with cDC2s plus IL-2
Supplementary Figure S23. Adoptively transferred OT-1 T cells stimulated by peritoneal cDCs are not detected in gut tissues .
As in Fig. 5E, OT-1 T cells were stimulated by peritoneal cDC1s, cDC2s, or cDC2s with IL-2, fluorescently labeled, and mixed in a 1:1 ratio, followed by adoptive transfer into recipient mice. 3 hours after adoptive transfer, the percentage of OT-1 T cells were analyzed from small intestinal lamina propria (SI-LP), small intestinal intraepithelial layer (SI-IEL), colon lamina propria (Colon-LP), and colon intestinal intraepithelial layer (Colon-IEL), to determine the homing index (HI) in each tissue. Representative flow cytograms are from 2 independent experiments.
